# EDA ligand triggers plasma membrane trafficking of its receptor EDAR via PKA activation and SNAP23-containing complexes

**DOI:** 10.1186/s13578-023-01082-8

**Published:** 2023-07-10

**Authors:** Yuyuan Yao, Ruihan Yang, Jian Zhu, David Schlessinger, Jian Sima

**Affiliations:** 1 School of Basic Medicine and Clinical Pharmacy, China Pharmaceutical University, Nanjing, 210009 China; 2 Department of Psychology, Eastern Illinois University, Charleston, IL 61920 USA; 3 Laboratory of Genetics and Genomics, NIA/NIH-IRP, 251 Bayview Blvd, Room 10B014, Baltimore, MD 21224 USA

**Keywords:** EDA, EDAR, Membrane trafficking, HED, TNFR

## Abstract

**Background:**

Ectodysplasin-A (EDA), a skin-specific TNF ligand, interacts with its membrane receptor EDAR to trigger EDA signaling in skin appendage formation. Gene mutations in EDA signaling cause Anhidrotic/Hypohidrotic Ectodermal Dysplasia (A/HED), which affects the formation of skin appendages including hair, teeth, and several exocrine glands.

**Results:**

We report that EDA triggers the translocation of its receptor EDAR from a cytosolic compartment into the plasma membrane. We use protein affinity purification to show that upon EDA stimulation EDAR associates with SNAP23-STX6-VAMP1/2/3 vesicle trafficking complexes. We find that EDA-dependent PKA activation is critical for the association. Notably, either of two HED-linked EDAR mutations, T346M and R420W, prevents EDA-induced EDAR translocation; and both EDA-induced PKA activation and SNAP23 are required for Meibomian gland (MG) growth in a skin appendage model.

**Conclusions:**

Overall, in a novel regulatory mechanism, EDA increases plasma membrane translocation of its own receptor EDAR, augmenting EDA-EDAR signaling in skin appendage formation. Our findings also provide PKA and SNAP23 as potential targets for the intervention of HED.

**Supplementary Information:**

The online version contains supplementary material available at 10.1186/s13578-023-01082-8.

## Background

Ectodermal Dysplasias (EDs) are a diverse group of inherited disorders that affect the morphogenesis of skin appendages including hair, teeth and several exocrine glands [1, 2]. Clinically, ED is classified into Anhidrotic/Hypohidrotic ED (A/HED) and Hidrotic ED depending on the presence of sweating, with most cases being anhidrotic [3, 4]. Although standard signaling pathways such as WNT, SHH, BMP and NOTCH all participate in regulating skin appendage development [5, 6], Ectodysplasin-A (EDA) is a skin-specific hormone required to promote the formation of distinct nascent skin appendages. Gene mutations in this pathway are usually not lethal and account for more than 90% of HED, characterized by malformation of hair follicles (HFs), sweat glands (SWs), teeth and Meibomian glands (MGs) [1]. Notably, a family with an X-linked ED skin phenotype and male-specific inheritance pattern were first reported by Charles Darwin in 1868 [7]; but it required over a century for three genes to be identified—X-linked EDA, EDAR, and EDARADD—that form a specific TNF ligand-receptor-adaptor family restricted to skin appendage formation [8–10].

The *ectodysplasin* gene encodes two closely related splice variants, EDA-A1 and EDA-A2, with the latter lacking only two amino acids: Glu308 and Val309. Interestingly, its receptor EDAR exclusively binds the EDA-A1 isoform (now named EDA), whereas another receptor XEDAR specifically binds EDA-A2 [11, 12]. The function of XEDAR and EDA-A2 remain obscure, while EDAR has been demonstrated to mediate EDA signaling by recruiting the complex of EDARADD/TRAF6/TAK1/TAB2 to activate NF-κB for gene transcription [4]. However, EDA activates a gene expression profile that is distinct from that induced by classic TNF ligands [13]. Such selectivity of gene expression can be partially explained by recent findings that suggest interaction of EDA-mediated NF-κB activation with a distinct chromatin remodeling SWI/SNF (BAF) complex to facilitate the selective gene expression [14]. Other studies further imply that EDA promotes the expression of specific chemokines [15] that could further induce cell migration for hair placode formation [16].

Thus far, studies have primarily focused on signal transduction downstream of the EDA-EDAR complex; but the molecular mechanisms of synthesis and plasma membrane trafficking of EDAR remains unknown.

Here, we surprisingly find that EDA promotes recruitment of its own receptor EDAR from a cytosolic compartment (Cyt) into the plasma membrane (PM). Using protein affinity purification, keratinocyte/skin tissue cultures, and EDA knockout (^−/−^) mice, we report a regulatory cascade responsible for EDAR PM translocation induced by EDA. Our data further reveal a novel pathogenetic mechanism: the T346M mutation in EDAR, causative of HED, blocks its PM trafficking by disrupting SNAP23 recruitment.

## Materials and methods

### Mice

Male EDA knockout (KO) mice (GemPharmatech, Stock # T017710) were crossed with C57BL/6 J female mice to obtain EDA-KO homozygote and wild type (WT) progeny. Skin tissues were isolated under a dissecting microscope (Olympus) for culture or histological study. Mice were maintained in a 12 h light/dark switching room at the temperature of 25 ± 2 °C, 70% relative humidity, with free access to food and water. All experiments were conducted in accordance with the Regulations for the Administration of Laboratory Animals in China and were approved by the Animal Ethics Committee of China Pharmaceutical University. Mice were genotyped using a Kit (Vazyme) according to the manufacturer's manual.

### cDNA, plasmids and stable cell lines

The EDA-A1 expression plasmid was kindly provided by Dr. David Schlessinger. Human EDAR, XEDAR, TNFR1 cDNAs (MiaoLing Plasmid Platform, China) were amplified by PCR, fused with EGFP at C-terminal, and cloned into the pHAGE-MCS-3 × HA (MiaoLing). The stable HaCaT cell lines expressing GFP only, EDAR-GFP, XEDAR-GFP and TNFR1-GFP were generated by a lentiviral transduction approach. Briefly, the pHAGE plasmids were dissolved in medium with the lentiviral packaging plasmid psPAX2 and the envelope protein plasmid pMD2G in proportion (1:1.5:1), mixed (total 24 µg plasmids) and then added to 36 µL PEI (1 mg/mL) transfection reagent (MCE), mixed well, and added to HEK293T cells cultured in a 10 cm dish. After 48 h transfection, the supernatant was collected in a sterile 15 mL centrifuge tube for centrifugation 5 min at 2000 rpm to remove cell debris and obtain the lentiviral particles. HaCaT cells in a 35 mm dish were transduced with lentiviral particles and cultured for 72 h, followed by digestion and dilution into 96-well plates as single cell cultured in each well. The successfully stable cell clones were screened with 4 μg/mL of puromycin (Thermo) for 48 h and then cells were maintained with 0.5 μg/mL of puromycin.

### Cell culture, transient transfection and conditioned medium (CM) collection

HaCaT or HEK293 cells were cultured in DMEM containing 10% FBS and maintained in an incubator containing 5% CO_2_ at 37 °C. Transient transfection in HaCaT or HEK293 cells with plasmids or SNAP23 siRNA were carried out using Lipo8000™ reagent (Beyotime) by following the manufacturer’s instruction. EDA-A1 CM was prepared as described previously [14]. Briefly, HEK293 cells (2 × 10^8^) were transfected with 100 µg of EDA-A1 expressing plasmid for 16 h and then changed to culture in DMEM containing 1% FBS. After 24 h, the medium was collected and concentrated tenfold using a Centricon Plus-10 filter (Millipore) and stored at − 80 °C until use.

### Site-directed mutagenesis and plasmid construction

Site-directed mutagenesis in EDAR cDNA were performed using an overlap-extension PCR protocol [17]. In brief, two DNA fragments were amplified by PCR with overlapping ends using WT EDAR-GFP cDNA as a template, then annealed and elongated into a full-length cDNA. Each nucleotide mutation in EDAR cDNA identified in HED patients was introduced by designed PCR primers. All primers for point mutations were listed in Supplementary Table 3. All the 6 mutated EDAR-GFP cDNAs were cloned into pHAGE-MCS-3 × HA plasmid and then transient transfected into HaCaT cells using Lipo8000^™^ (Beyotime). The WT EDAR and T346M mutant were also cloned into modified pAAV-CAG-GFP plasmid with additional T2A motif (Additional file 1: Fig. S8) for further study.

### Immunoblotting and immunofluorescence (IF)

For immunoblotting, equal amounts of protein of each sample were prepared and separated by SDS-PAGE and transferred onto the nitrocellulose (NC) membranes. The membrane was blocked with 5% no fat milk in PBST at room temperature for 1 h and incubated with the indicated antibodies overnight at 4 °C, and then with HRP Goat Anti-Mouse IgG (H + L) or HRP Goat Anti-Rabbit IgG (H + L) secondary antibody for 1 h at room temperature. The samples were visualized with High-signal ECL Western Blotting Substrate (Tanon) and Fully Automatic Chemiluminescence Image Analysis System (Tanon, 4600SF). For IF, cells were cultured on coverslips and fixed in 4% PFA for 30 min, permeabilized for 15 min in 0.3% Triton X-100 on ice, and blocked with 3% BSA for 1 h. Then the cells were incubated with primary antibodies overnight at 4 °C. After washed with PBS for 3 times, cells were exposed to secondary antibodies (Thermo) for 1 h. Then cells were washed with PBS for 3 times and mounted with Pro-Long^™^ Gold Antifade with DAPI. Images were collected with a fluorescence microscope (Olympus, IX73). The ratio of fluorescent intensity was measured using ImageJ 1.53 software. All antibodies used in this study were listed in Supplementary Table 2.

### Preparation of protein lysate

The cells were lysed in a lysis buffer (0.5% NP40, 350 mM NaCl, 20 mM HEPES, complete cocktail), and then centrifuged at 2000 rpm for 5 min at 4 °C. The supernatant was collected as the cytoplasmic lysate. The precipitate was washed twice with cold PBS, followed by centrifugation at 15,000 rpm for 10 min to obtain a plasma membrane component, which was further lysed with 5 M guanidine hydrochloride and then centrifuged at 15,000 rpm for 10 min. After that, the supernatant was transferred to a dialysis tube (Thermo, Cat. 69,562) and dialyzed in PBS overnight at 4 °C. After that, liquid was then collected as plasma membrane lysate. For total cell lysate collection, cells were lysed with a 2 × sample buffer (20 mM dithiothreitol, 6% SDS, 0.25 M Tris, pH 6.8, 10% glycerol, 10 mM NaF and bromophenol blue) at approximately 1 × 10^7^ cells per mL, followed by 5 min heating in boiling water bath. After that, the protein extracts were sonicated for 10 s 4 times and then centrifuged at 15,000 rpm for 10 min to collect supernatant as total cell lysate.

### Gel filtration, immunoprecipitation (IP), and mass spectral (MS) analysis

Superpose 6 gel-filtration analysis, were performed as previously described [14]. Briefly, protein extracts (16 mg) was directly applied to a Superose 6 column (HR 16/50; GE) equilibrated with the column running buffer containing 20 mM HEPES (pH 7.9), 200 mM NaCl, 1 mM DTT, 0.1 mM PMSF, and 10% glycerol. Fractions were collected (1.5 mL each) and analyzed by 10% SDS-PAGE and immunoblotting. EDAR-containing protein complex was immunopurified with anti-Flag M2 affinity gel (Sigma-Aldrich) and anti-HA affinity gel (Cell Signaling Technology) from HaCaT extracts by using an IP protocol. Briefly, 1 mL (8 mg/mL) of protein extracts were diluted 10 times with IP buffer (20 mM HEPES [pH 7.9], 200 mM NaCl, 1 mM dithiothreitol [DTT], 0.2 mM PMSF, 10% glycerol) and incubated with 100 μL of anti-Flag or anti-HA gels for overnight at 4 °C. The immunoprecipitate was washed 4 times with the IP buffer. The complex on the beads was eluted from the beads by using 100 mM glycine–HCl buffer (pH 2.5). The eluted complex was subjected to SDS-PAGE and immunoblotting analysis. Less than 10% of the input was loaded as controls for immunoblotting. For mass spectrometric analysis, the eluted proteins were shipped frozen on dry ice to The Taplin Biological Mass Spectrometry Facility (Harvard University).

### Preparation of adeno-associated viral (AAV) particles

The pAAV-CAG-GFP and pAAV-EGFP-U6-shRNA plasmids were purchased from Addgene. The human EDAR cDNAs and mutants described above was inserted into pAAV-CAG-GFP at downstream of T2A motif. A shRNA sequence targeting mouse SNAP23 (5′-GGAAGAGAACCTGACTCAA-3′) was cloned into pAAV-ZsGreen1-shRNA vector (MiaoLing) (Additional file 1: Fig. S8). The structures of AAV plasmids used in this study were listed inAdditional file 1: Fig. S8. AAV production was performed as AAV5 stereotype using a protocol described by Addgene (https://www.addgene.org/protocols/aav-production-hek293-cells/).

### Eyelid organotypic culture

The eyelid organotypic culture assays were conducted as described [14]. Briefly, the eyelid skins of E15.5 mouse embryos were isolated under a dissecting microscope and then washed twice with cold PBS and placed on 0.4 µm Millicell culture insert (Millipore) and cultured in DMEM with 10% FBS. For gene transfection, AAV5 particles expressing shRNA against SNAP23 or over-expressing EDAR WT or mutants (1 × 10^8^ GC/mL) were added into the medium. For PKA inhibitor/agonist treatment, H89 was used at 60 μM and Forskolin was used at 20 μM. After 48 h, the eyelids were fixed and paraffin-embedded for histological study. The longest MG germs from serial sections were measured, and the distance between epidermal basal cell edge and the outer most edge of germs was calculated. A total of 10–15 MG germs were measured and quantified from each cultured eyelid tissue.

### Histology and immunohistochemistry (IHC)

For histological analyses, eyelids from mice at each time point were fixed in 10% formaldehyde and embedded in paraffin and 5 µm sections were then cut using a microtome (Leica). For IHC, eyelid skin fixed in 10% formaldehyde was dehydrated and embedded in paraffin (Sigma). 5 μm sections were cut, deparaffinized and ‘unmasked’ by heating at 121 °C for 2 min in an Antigen Unmasking Solution (H-3300, Vector Laboratories). Histological immunofluorescence staining was carried out with primary and secondary antibodies as listed (Additional file 1: Table S2). Sections were blocked with a serum-free protein blocking solution (X0909, Dako) for 30 min before antibody application, and antibodies were diluted in an Antibody Dilution solution (S0809, Dako). Tissue slices were mounted with Pro-Long^™^ Gold Antifade with DAPI and fluorescence images were captured by a fluorescence microscope (Olympus, IX73).

### Data and statistical analyses

All data were expressed as mean ± SD. Student’s *t*-test was used to identify statistically significant differences between groups. GraphPad Prism 8.0 was used for the statistical analysis. A *P* value < 0.05 was considered statistically significant.

## Results

### EDA triggers plasma membrane translocation of its receptor EDAR

**Fig. 1:**
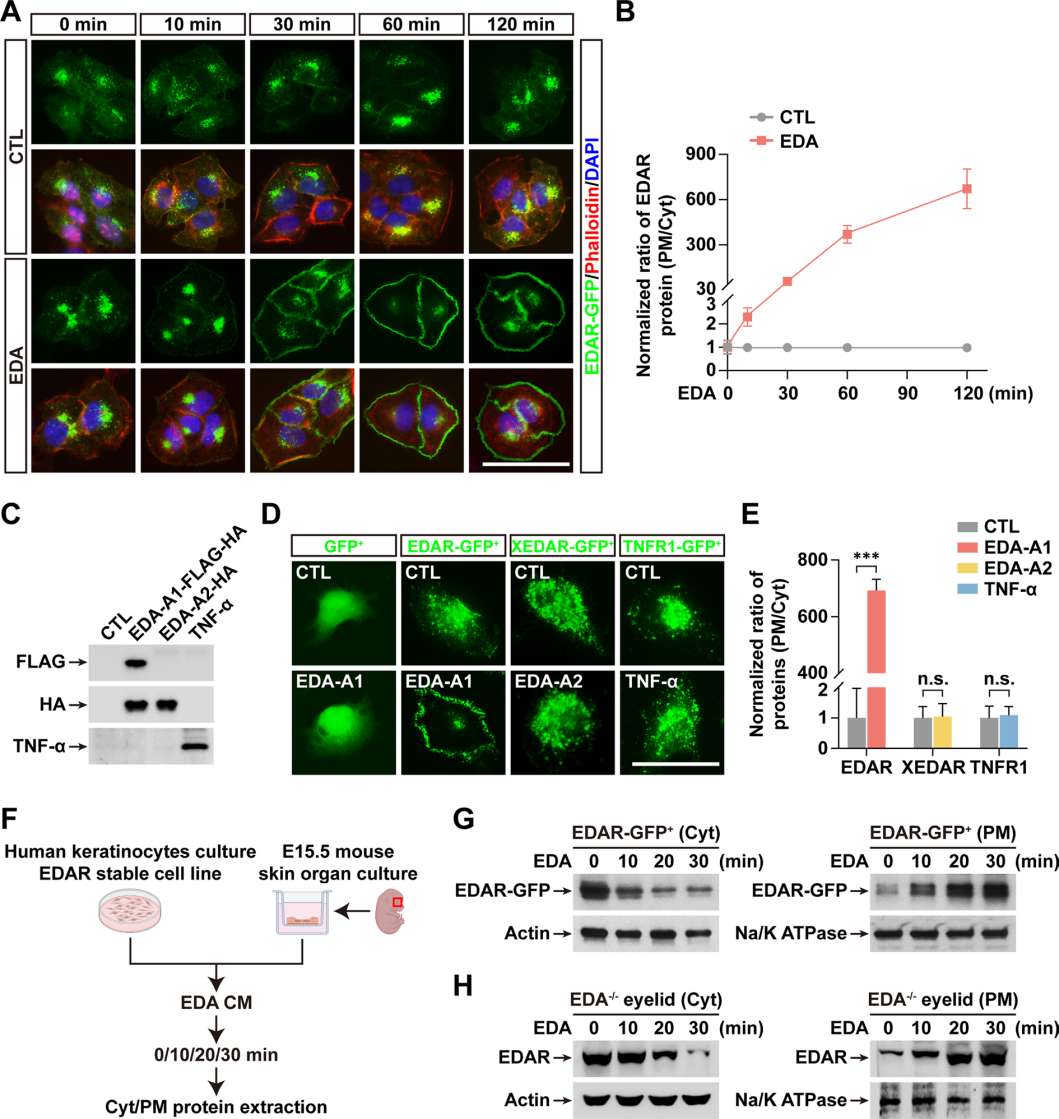
EDA triggers PM translocation of its receptor EDAR. **A** Fluorescence images of EDAR-GFP (green) in EDAR-GFP^+^ cells are shown with treatment of EDA-A1 (EDA) conditioned medium (CM) at indicated time points. Cytoskeleton labeled with phalloidin (red). Nuclei counterstained with DAPI (blue). **B** Quantification shows the ratio of EDAR levels in plasma membrane (PM) to cytosolic compartment (Cyt) at each indicated time points in **A**. The ratio of PM/Cyt at 0 min was normalized to 1.0. In each condition, at least 10 random images (each image includes ≥ 3 cells) from 3 independent experiments were used for quantification. **C** Immunoblotting shows the successful expression of EDA-A1, EDA-A2 and TNF-α in CM. Medium collected from naïve HEK293 cells as control (CTL) CM. **D** Fluorescence images (green) show the cellular location of GFP, EDAR-GFP, XEDAR-GFP, or TNFR1-GFP treated with indicated CM containing EDA-A1, EDA-A2, or TNF-α. **E** Quantification shows the PM/Cyt ratio of EDAR, XEDAR and TNFR1 in cells with indicated treatment as in **D**. Data from at least 10 random images (each image includes ≥ 3 cells) in each condition from 3 independent experiments. **F** A diagram shows the workflow using EDAR-GFP^+^ cell culture and mouse eyelid organotypic culture with indicated EDA treatment. **G**–**H** Immunoblotting shows the levels of EDAR in Cyt (left) and PM (right) from EDAR-GFP^+^ cells **G** or EDA knockout (^−/−^) eyelid cultures **H**. Actin or Na/K ATPase as loading control for Cyt or PM. Error bars indicate mean ± SD from at least 30 cells from 3 independent experiments. ***P < 0.001; *n.s*, not significant; Student’s *t*-test. Scale bar, 20 μm

To study the interplay of EDA and its receptor EDAR, we sought to use a cell-based model to observe the dynamics of EDA-EDAR interaction. We first generated a derivative of the HaCaT human keratinocyte stable cell line (“EDAR-GFP^+^”) that expresses EDAR fused to a green fluorescent protein (GFP) tag at its C-terminal. We then unexpectedly found that the majority (~ 92.3%) of EDAR-GFP was located in a cytosolic compartment (Cyt) surrounding cell nucleus (Fig. 1A), likely a perinuclear region suggested as an important area for protein trafficking [18, 19]. We then tested whether extracellular ligand EDA could affect the cellular location of its membrane receptor EDAR by adding conditioned medium (CM) containing active EDA protein to the cultures. Indeed, EDA rapidly and strongly induced PM translocation of EDAR (Fig. 1A). Other 2 clones of EDAR-GFP^+^ stable cell lines showed an identical phenotype (Additional file 1: Fig. S1). Fluorescence images showed that 10 min stimulation with EDA initiated pronounced movement of Cyt EDAR to the cell PM. Quantification of fluorescence intensity demonstrated time-dependent PM translocation of EDAR within 2 h (Fig. 1B). Notably, after a 2 h treatment of EDA, ~ 98.3% of EDAR was relocated from Cyt into PM, and the ratio of EDAR (PM/Cyt) was thereby increased by ~ 670-fold compared to PBS treatment (Control, CTL) (Fig. 1B). Unlike EDAR, neither XEDAR nor TNFR1—the other two TNFR member proteins—showed such ligand-triggered PM translocation of its own receptor (Fig. 1C–E). To exclude the possible clone-specific effect, we used other two clones of stable cell lines expressing each EDAR-GFP, XEDAR-GFP or TNFR1-GFP and found a same cell behavior (Additional file 1: Fig. S1). Thus, these data suggest a distinct regulatory consequence of EDA-EDAR interaction that might augment the activity of EDA signaling in skin appendage formation.

We then fractionated cell contents to recover the PM and cytoplasmic proteins (including vesicles and endosomes) and assessed the levels of EDAR in each fraction either from HaCaT cell or from skin organotypic cultures of EDA^−/−^ eyelids (Fig. 1F). Immunoblotting data confirmed the phenotype of EDA-induced translocation of EDAR from Cyt to PM in both systems (Fig. 1G, H, and Additional file 1: Fig. S2). Total EDAR protein did not change for at least 2 h after EDA treatment (Additional file 1: Fig. S3), suggesting that the promotion of its synthesis during longer duration of EDA stimulation [20] did not contribute to the higher levels of PM-associated EDAR. In agreement with EDAR-GFP translocation (Fig. 1A, B) as the underlying mechanism, PM protein levels of EDAR rapidly increased while Cyt EDAR decreased during 10 to 30 min of treatment of EDA (Fig. 1G, H and Additional file 1: Fig. S2).

In sum, EDA triggers rapid cell surface translocation of its own receptor EDAR from a cytosolic compartment in both HaCaT cells and skin tissues.

### EDA induces the recruitment of AKAP9 and SNAP/STX/VAMP trafficking proteins to EDAR

**Fig. 2:**
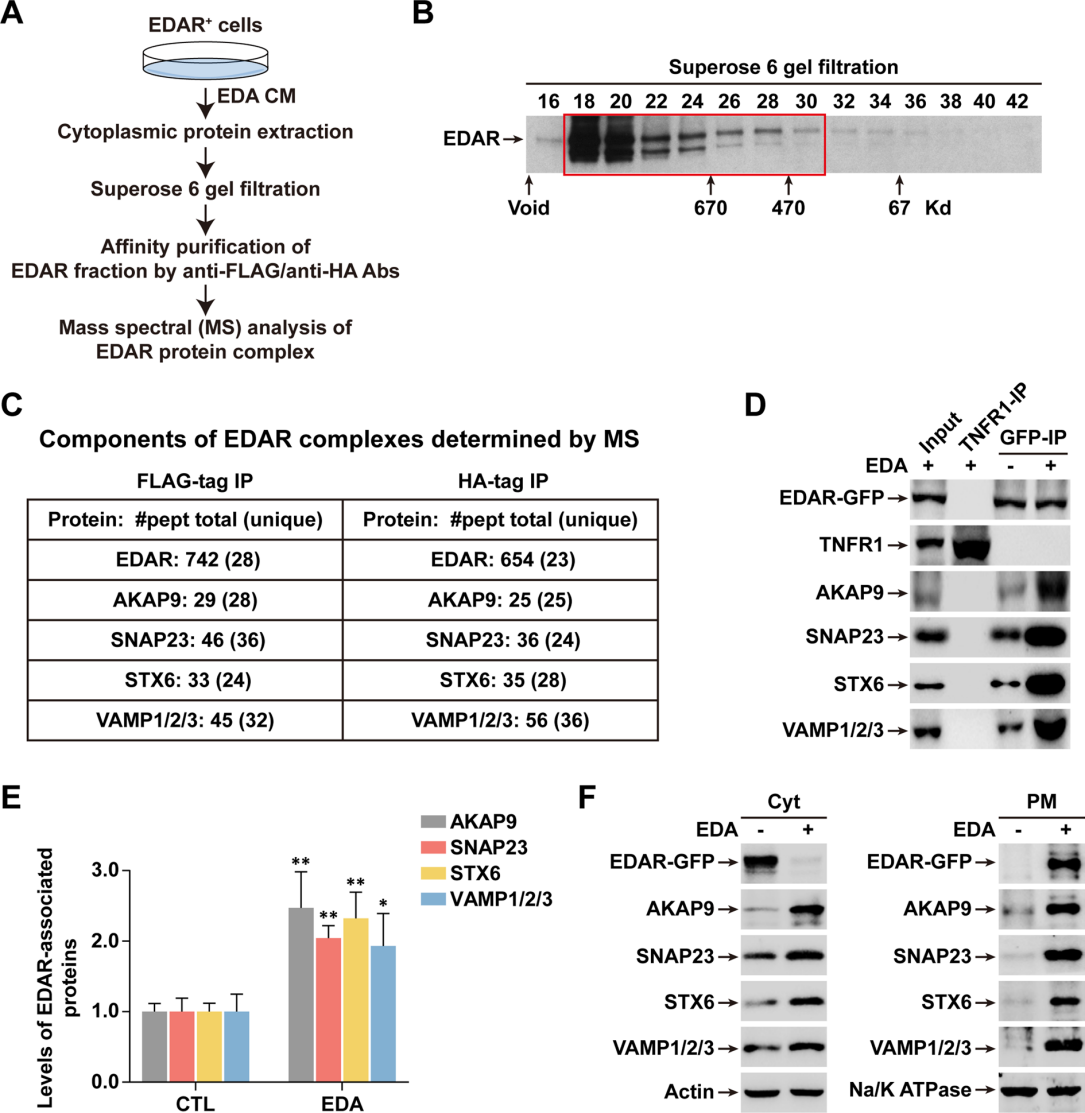
Protein complex purification reveals the binding partners of EDAR. **A** A schematic shows the workflow of EDAR complex purification. **B** Immunoblotting shows gel-filtration profiles in the cytoplasmic protein extracts from EDAR^+^ cells. The red rectangle indicates fractions collected for immunoprecipitation (IP) and mass spectral (MS) analysis. **C** Key components of EDAR complexes determined by MS. The numbers (#) of total and unique peptides (pept) of indicated proteins are shown. **D** GFP-IP with cytoplasmic protein extracts was followed by immunoblotting with the indicated antibodies. 5% cytoplasmic extracts from EDAR-GFP^+^ cells used as input. IP with TNFR1 antibody as the negative control. **E** Quantification of the levels of EDAR-associated proteins in **D**. The protein levels with no EDA treatment were normalized to 1.0. Data from 3 independent experiments. **F** Immunoblotting shows the levels of indicated proteins in Cyt or PM fraction with (+) or without (−) EDA treatment. Error bars indicate mean ± SD. *P < 0.05, **P < 0.01; Student’s *t*-test

To investigate the mechanism of EDAR PM translocation, we adapted an affinity-purification approach that we had previously used [14] to recover intracellular EDAR complexes from HaCaT keratinocytes after EDA stimulation (Fig. 2A). We first made a derivative of the HaCaT stable cell line (EDAR^+^) expressing the EDAR fused to a Flag tag followed by another HA tag at its C-terminal. Immunofluorescence (IF) images using anti-Flag and anti-HA antibodies confirmed the EDA-dependent PM translocation of EDAR (Additional file 1: Fig. S4). We then fractionated cytoplasmic extracts from EDAR^+^ cells that had been treated with EDA CM for 2 h. After Superose 6 gel-filtration chromatography, peak fractions containing EDAR protein complexes (Fig. 2B) were collected and immunoprecipitated (IP) with anti-Flag and anti-HA affinity-gels. Mass spectral (MS) analyses (Fig. 2C and Additional file 1: Table S1) showed the expected presence of EDAR in both Flag and HA immunoprecipitates to a similar extent. But interestingly, abundant peptides of trafficking proteins including SNAP23, STX6 and VAMP1/2/3 were also observed. This suggested that these major trafficking proteins were involved in EDAR PM translocation. In addition, AKAP9, an adaptor protein binding to protein kinase K (PKA), was also highly enriched in both immunoprecipitates (Fig. 2C and Additional file 1: Table S1).

IP using cytoplasmic extracts then revealed that increased levels of all these proteins were recruited to associate with intracellular Cyt EDAR after EDA treatment (Fig. 2D), with an increase of ~ 2.5-fold AKAP9, ~ 2.0-fold SNAP23, 2.3-fold STX6 and 1.9-fold VAMP1/2/3 (Fig. 2E). IF data further confirmed the colocalization of EDAR with these trafficking proteins under EDA treatment (Additional file 1: Fig. S5). Immunoblotting using total cell lysate also showed elevated levels of these trafficking proteins upon EDA treatment (Additional file 1: Fig. S6). We next assessed the trafficking protein content in both cytoplasmic and PM fractions and found a mild increase of each of them in the cytoplasmic fraction along with a sharp increase in the PM fraction (Fig. 2F), suggesting that these trafficking proteins were not only activated to attach to EDAR for PM trafficking, but that their expression levels were also upregulated by EDA stimulation.

We infer that EDA induces the recruitment of PKA adaptor AKAP9 and trafficking proteins to EDAR, an association likely required for the PM translocation of EDAR.

### PKA activity and SNAP23 are both indispensable for EDAR PM translocation

**Fig. 3:**
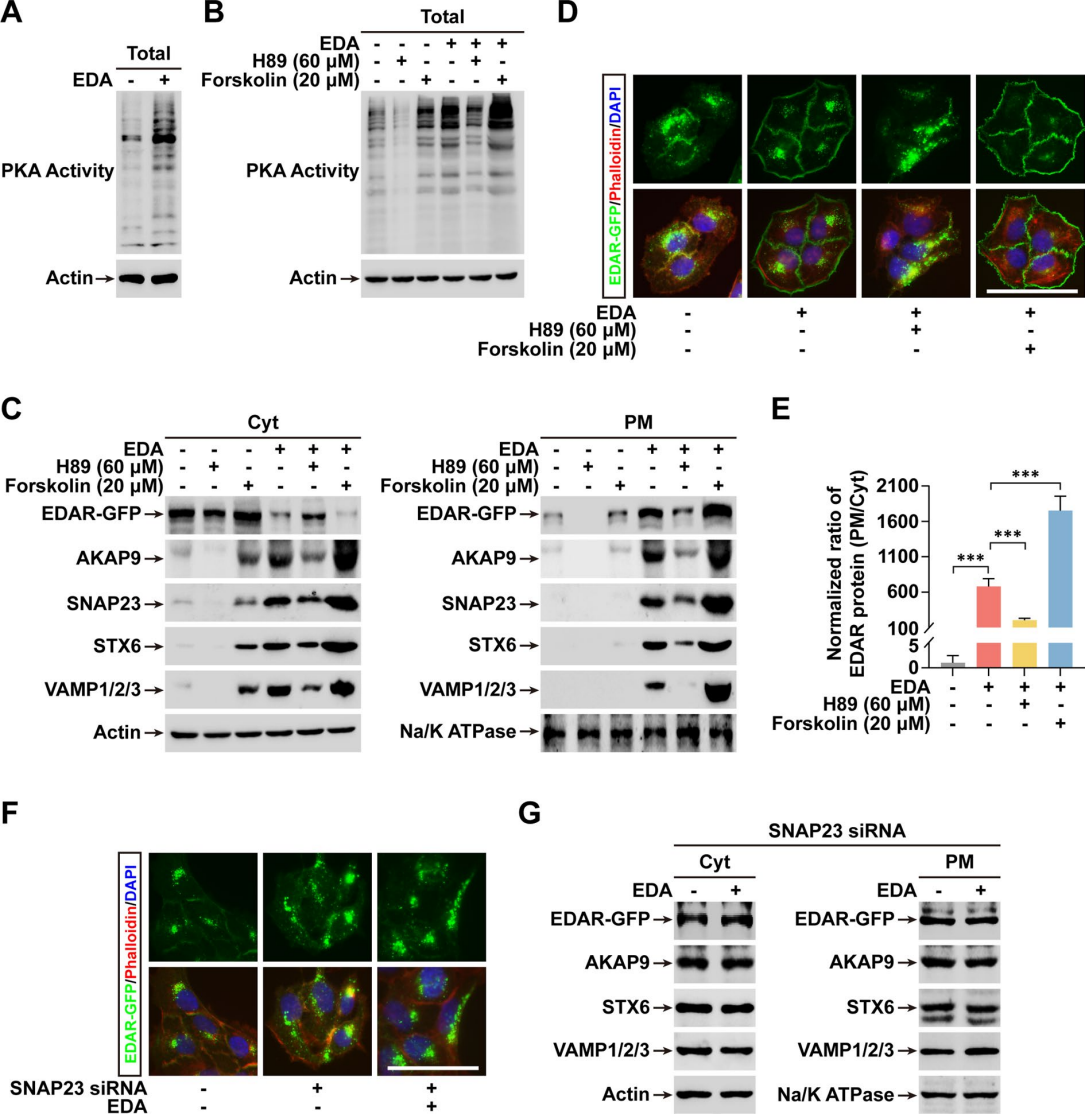
EDAR PM trafficking requires PKA activation and SNAP23. **A**–**B** Immunoblotting shows the intensity of phosphorylated PKA substrates (PKA activity) with or without treatment of EDA, H89 (60 μM), or Forskolin (20 μM). **C** Immunoblotting shows the levels of indicated proteins in Cyt or PM fraction with or without treatment of EDA, H89 or Forskolin. **D** The localization of EDAR-GFP in EDAR-GFP^+^ cells with or without indicated treatment of EDA, H89 or Forskolin. **E** Quantification shows the ratio of EDAR levels in PM to Cyt in **D**. Data from at least 10 random images in each condition from 3 independent experiments. **F** As in (D), except with indicated treatment of EDA and transfection of SNAP23 siRNAs. **G** As in **C**, except with indicated treatment of EDA and transfection of SNAP23 siRNAs. Error bars indicate mean ± SD from at least 30 cells from 3 independent experiments. ***P < 0.001; Student’s *t*-test. Scale bar, 20 μm

Its circumstantial association suggested that PKA activity might be essential for EDA-triggered EDAR movement to the cell membrane. Using an antibody against phospho-(Ser/Thr) PKA substrates, we examined the PKA activity in EDAR-GFP^+^ cells before and after EDA treatment. Immunoblotting showed that EDA indeed markedly increased PKA activity (Fig. 3A). An inhibitor H89 and an agonist Forskolin of PKA (Fig. 3B) were further tested in EDAR-GFP^+^ cell cultures; consistent with the obligate requirement of PKA in the process, H89 completely blocked and Forskolin augmented the PM sorting of EDAR (Fig. 3C, D).

We next evaluated the effect of PKA activity on trafficking proteins. Immunoblotting showed that upregulated PKA activation also strongly increased the levels of EDAR-associated proteins in both Cyt and PM fractions when treated with EDA, while inhibition of PKA activity by H89 showed the opposite effect (Fig. 3E).

To illustrate the role of SNAP23, the SNAP protein attached to EDAR (Fig. 2C), we transfected siRNAs against SNAP23 in EDAR-GFP^+^ cells and assessed EDAR trafficking. The siRNA transfection effectively downregulated SNAP23 but showed no effect on trafficking proteins in EDAR complexes (Additional file 1: Fig. S7). Strikingly, knockdown (KD) of SNAP23 entirely prevented the EDA-dependent movement of EDAR from Cyt to PM (Fig. 3F) and failed to recruit other proteins of trafficking complexes (STX6 or VAMP1/2/3) to their target EDAR (Fig. 3G).

Taken together, our results suggest that EDA-induced PM movement of EDAR requires both AKAP9-mediated PKA activation and SNAP23-dependent involvement of STX6-VAMP1/2/3 trafficking complexes.

### Inhibition of PKA or SNAP23 impedes EDAR PM translocation and creates a phenocopy of EDA^−/−^ mice in skin appendage formation

**Fig. 4:**
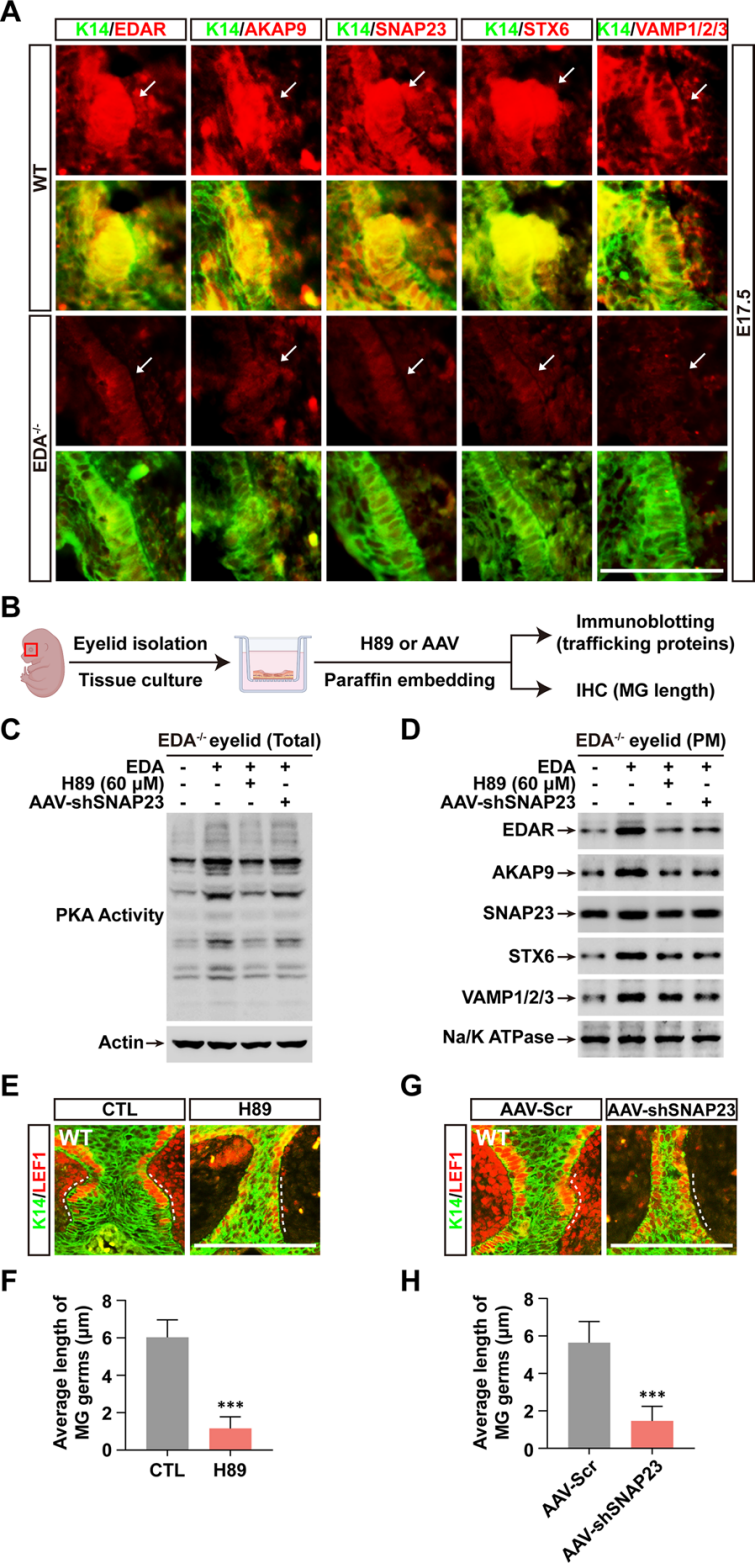
PKA activation and SNAP23 are both required for EDA-mediated MG growth. **A** Immunohistochemical (IHC) staining shows the expression of K14 (green) and other indicated proteins (red) in WT or EDA^−/−^ eyelids at embryonic day 17.5 (E17.5). Arrows indicate MG germs. Scale bar, 10 μm. **B** A graphic drawing shows the workflow of eyelid organotypic culture followed by indicated treatment and immunoblotting / IHC study. **C** Immunoblotting shows the intensity of PKA activity with indicated treatment of EDA, H89, or AAV-mediated SNAP23 KD (shSNAP23) in EDA^−/−^ eyelid cultures. **D** Immunoblotting shows the levels of indicated proteins in PM fraction from identical tissue cultures and treatment as in **C**. **E**–**F** IHC shows the expression of K14 (green) and LEF1 (red) after 2 days culture of WT eyelids isolated at E15.5. MG germs indicated by dotted lines. The MG length were calculated as described (see Methods) and quantified as shown in **F**. **G**–**H** As in **E** and **F**, except with transfection of AAV-scramble (Scr) or AAV-shSNAP23. Error bars indicate mean ± SD from at least 20 MGs in total 3 independent experiments. ***P < 0.001; Student’s *t*-test. Scale bar, 20 μm

EDA signaling initiated by EDA binding to EDAR is a distinct morphogenetic pathway responsible for skin appendage formation. We therefore asked whether the mechanism of EDAR PM translocation also controls skin appendage growth. To test this notion, we used a model of Meibomian gland (MG) formation that we had established [14, 21]. Immunohistochemical (IHC) data showed lower expression of EDAR in MG germs of EDA^−/−^ mice (Fig. 4A), consistent with previous findings in other skin appendages [15, 20]. Like EDAR, AKAP9 and EDAR-associated trafficking proteins (SNAP23, STX6 and VAMP1/2/3) were all very low in MG germs (Fig. 4A).

Next, using the MG organotypic culture assay [21] and the workflow schematized in Fig. 4B to check for possible function of the EDAR-associated molecules. Immunoblotting indicated that H89 inhibited the PKA activation induced by EDA stimulation (Fig. 4C); and in accord with the results in cell cultures, the organotypic cultures of EDA^−/−^ eyelids showed that EDA-induced enrichment of AKAP9 and other trafficking proteins in the PM fraction was inhibited by either H89 or AAV5-mediated SNAP23 KD (AAV-shSNAP23) (Fig. 4D). Analyzing length, H89 sharply shortened MG germs by ~ 80.6% compared to wildtype (WT) (Fig. 4E, F), and SNAP23 KD had a similar reduction of ~ 74.1% (Fig. 4G, H). Consistent with the results in EDA^−/−^ eyelid cultures, either H89 or SNAP23 KD reduced EDAR PM translocation in WT cultures (Additional file 1: Fig. S9, Additional file 1: Fig. S10).

Thus, EDA-dependent PKA activation and SNAP23 recruitment to EDAR are both indispensable for skin appendage formation.

### Two HED-linked EDAR mutations block EDA-dependent PM translocation of EDAR

**Fig. 5:**
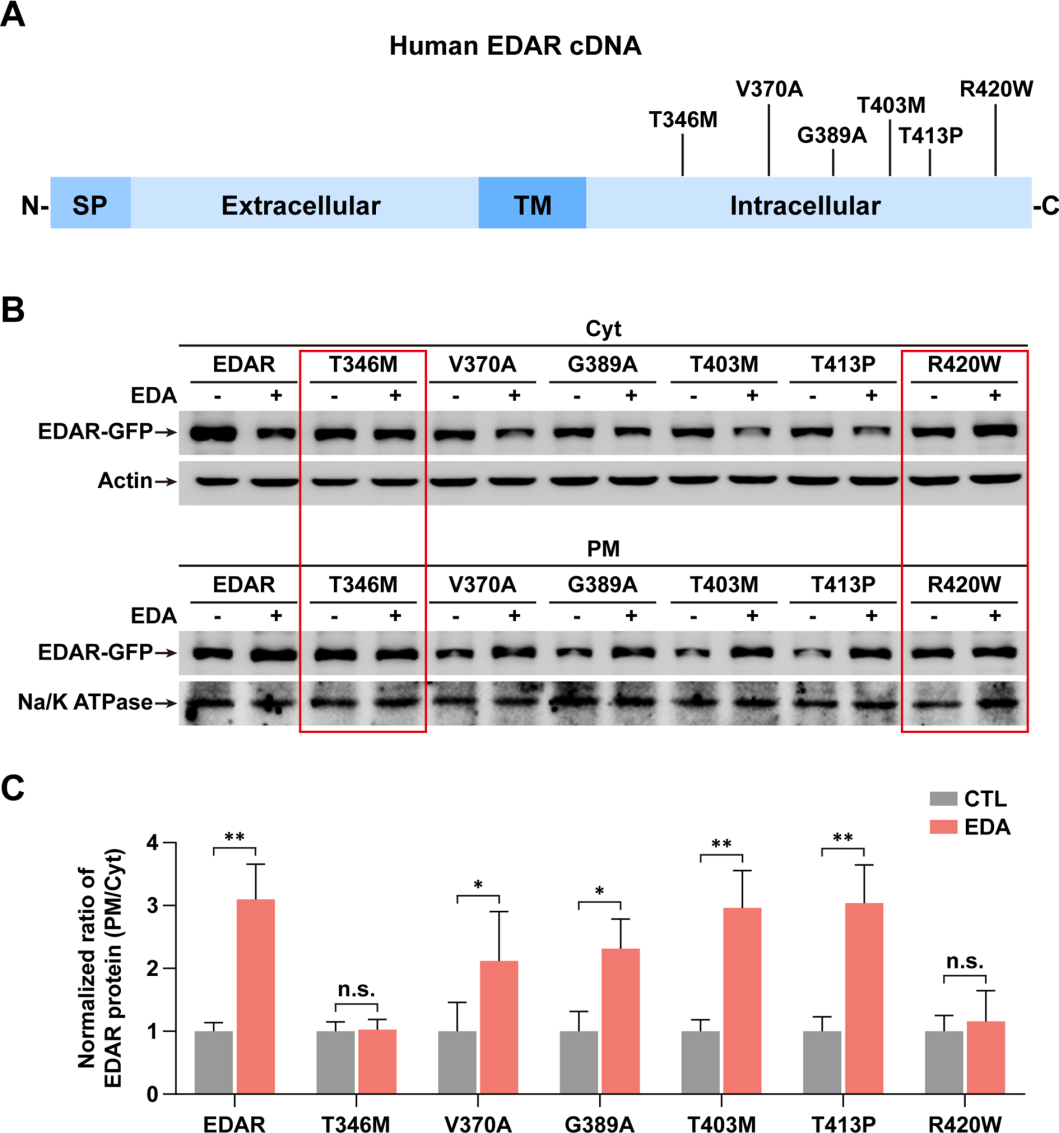
Screening identifies HED-linked mutations in EDAR leading to failed PM translocation. **A** A drawing structure of human EDAR including signal peptide (SP), extracellular, transmembrane (TM) and intracellular domains. Total six identified point mutations of EDAR exclusively at intracellular domain from HED patients are shown. **B** Immunoblotting shows the levels of EDAR in HaCaT cells transfected with plasmids expressing each indicated EDAR mutants. Red rectangles show T346M and R420W with undetectable changes. **C** Quantification of the levels of EDAR-associated proteins in **B**. The protein levels with no EDA treatment were normalized to 1.0. Data from 3 independent experiments. Error bars indicate mean ± SD. *P < 0.05, **P < 0.01, *n.s* not significant; Student’s *t*-test

We hypothesized that some gene mutations of EDAR identified in HED may affect its PM translocation induced by EDA stimulation. To test this hypothesis, we checked 6 EDAR mutations associated with strong HED phenotypes [22–26] and located within the intracellular domain of EDAR protein (so that they would not be likely to affect binding to extracellular EDA) (Fig. 5A). Using site-directed mutagenesis, we constructed plasmids expressing EDAR mutants including T346M, V370A, G389A, T403M, T413P or R420W. After transfection, EDA CM was added to cell cultures with each lesion to assess PM translocation of EDAR. Again, WT EDAR protein showed the clear movement from Cyt to PM after a 2 h treatment of EDA. But interestingly, two of the 6 EDAR mutants T346M and R420W, showed no PM translocation (Fig. 5B, C), which was further confirmed by fluorescence images of T346M and R420W (Additional file 1: Fig. S11). Because R420W also blocks the interaction between EDAR and EDARADD [22], we further focused on T346M.

### T346M diminishes EDAR function by inhibiting SNAP23 recruitment to EDAR

**Fig. 6:**
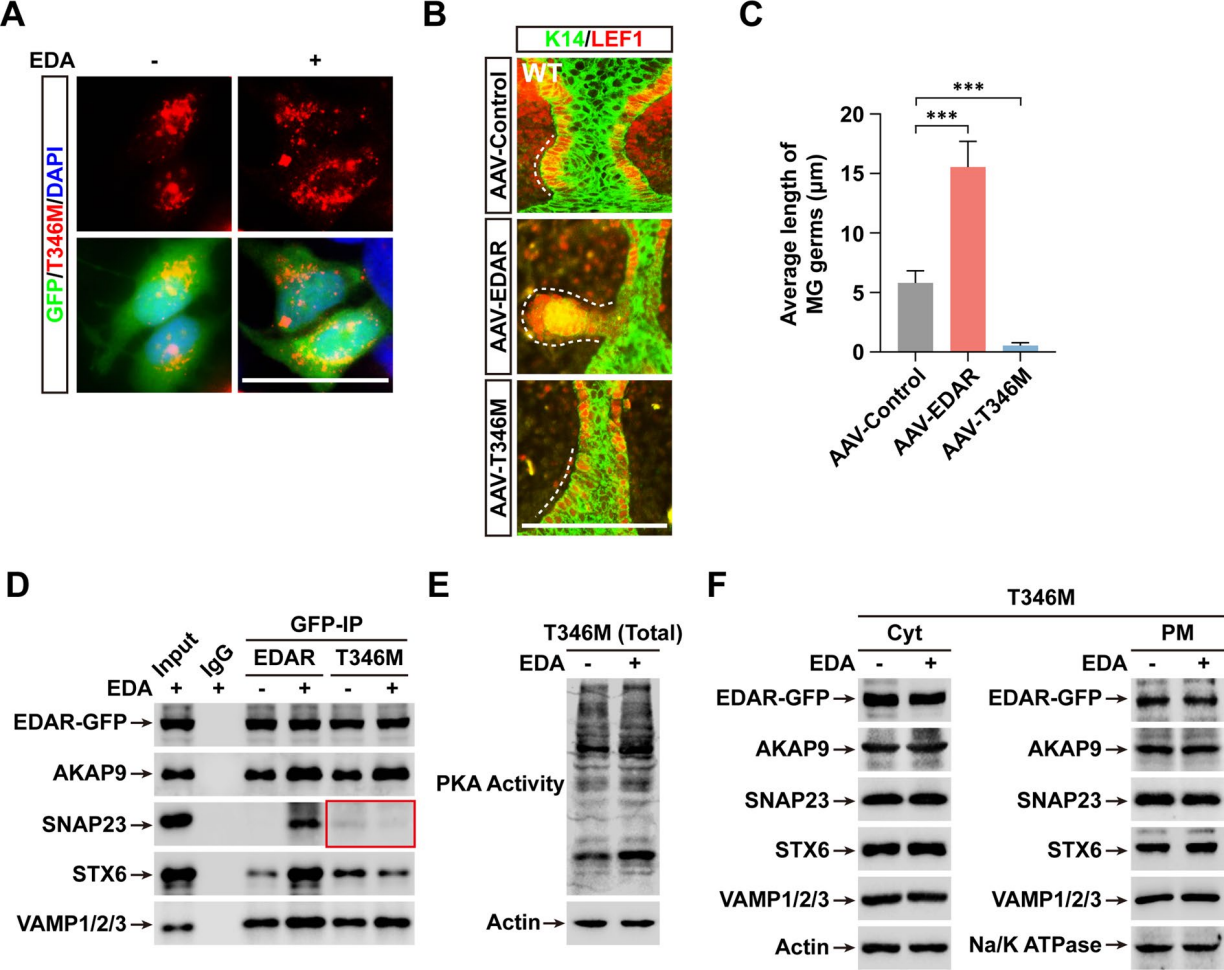
T346M blocks EDAR transportation and completely restrains MG growth. **A** Fluorescence images show the location of EDAR-T346M expressed by AAV particles also encoding GFP separated by T2A motif. **B** LEF1 (red) and K14 (green) IHC staining of cultured WT eyelids transduced by AAV particles encoding GFP, EDAR or T346M. **C** Quantitation of MG length in **B**. **D** GFP-IP was conducted using cytoplasmic lysates from cells treated with or without EDA and expression of WT, EDAR or T346M. Immunoblotting shows the protein levels using the indicated antibodies. Red rectangle shows SNAP23 with obvious changes. **E** Immunoblotting shows the intensity of PKA activity in cells expressing T346M with or without EDA treatment. **F** Immunoblotting images show the levels of indicated proteins in Cyt and PM fractions from cells expressing T346M with or without EDA treatment. Error bars indicate mean ± SD from at least 20 MGs in total 3 independent experiments. ***P < 0.001; Student’s *t*-test. Scale bar, 20 μm

To study the effect of the T346M mutation on EDAR function, we transduced HaCaT cells with AAV5 particles expressing T346M fused to an HA-tag at the C-terminal and checked the localization of T346M. As expected, IF of HA-tag showed that T346M was largely restricted to the Cyt, whether or not EDA was present (Fig. 6A). Next, we examined the effect of T346M on MG growth. As expected, MGs transduced with AAV5 particles expressing WT EDAR exhibited ~ 2.7-fold longer germs compared to those expressing only a GFP (control, CTL) (Fig. 6B, C). However, ectopic expression of T346M completely abolished the growth of MG germs (Fig. 6B, C), demonstrating that T346M is a loss-of-function mutation in EDA-dependent skin appendage formation.

To address how T346M mutation blocked EDAR trafficking, we used IP experiments to check for complex formation between EDAR and trafficking proteins. As shown in Fig. 6D, AKAP9 or VAMP1/2/3 binding to EDAR was not affected by the T346M mutation upon EDA stimulation, consistent with the increased PKA activity in T346M-expressing cells treated with EDA (Fig. 6E). But unlike AKAP9 action, EDA-induced recruitment of SNAP23 and STX6 was inhibited by the T346M mutation, with SNAP23 being the highly suppressed (Fig. 6D). We further examined the levels of EDAR and its associated components in Cyt and PM fractions and found that the T346M mutation indeed prevented the EDA-induced PM enrichment of EDAR and all its associated proteins (Fig. 6F).

In sum, HED-linked T346M, as a loss-of-function mutation, inhibits SNAP23 attachment and thus impairs the PM translocation of EDAR, which eventually results in the failure of normal skin appendage development.

### Either PKA activation or SNAP23 overexpression augments EDA-dependent MG growth

**Fig. 7:**
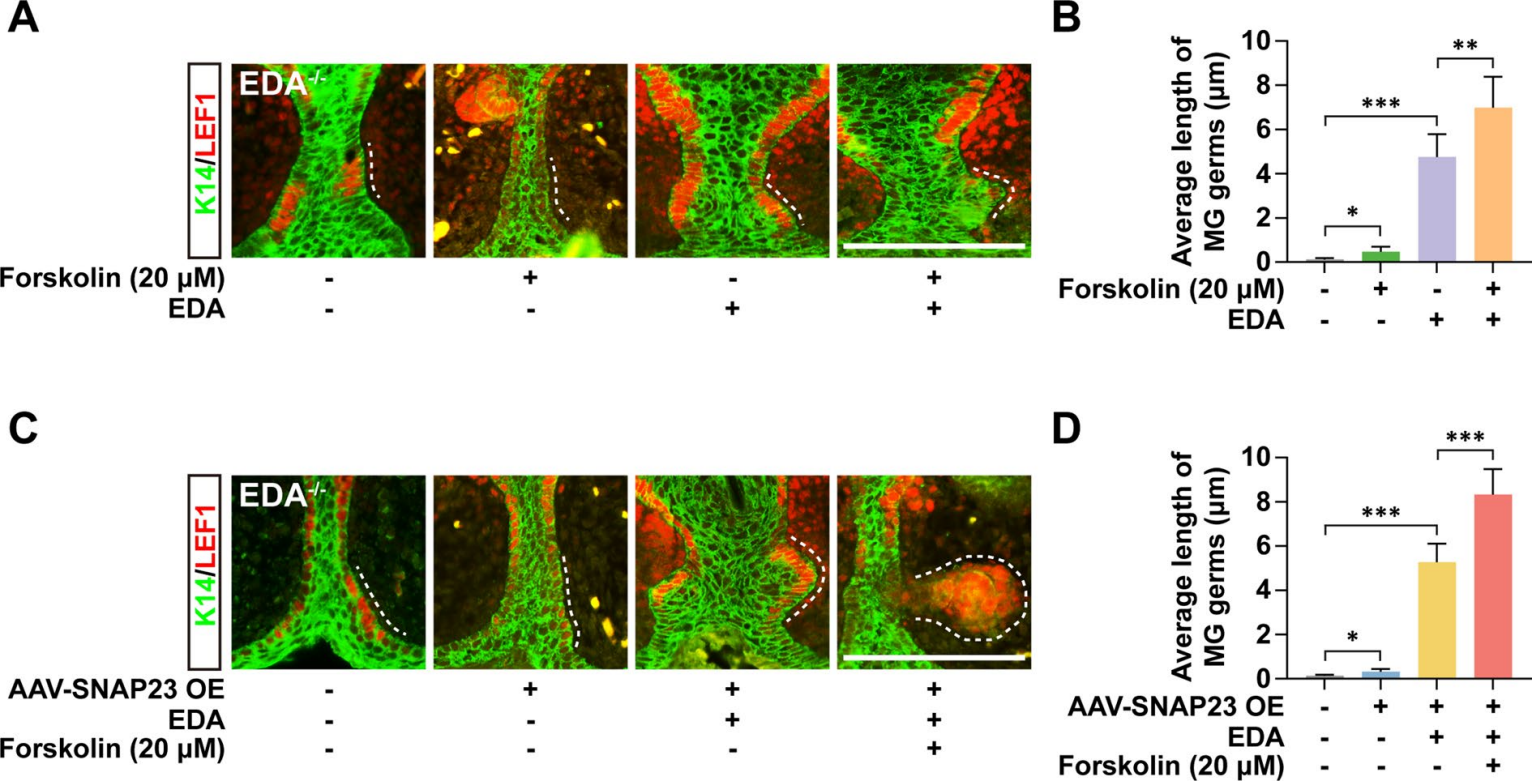
Either PKA activation or ectopic expression of SNAP23 augments EDA-dependent MG growth. **A**, **C** IHC staining of LEF1 (red) and K14 (green) in cultured EDA^−/−^ eyelids with indicated treatment. **B**, **D** Average length of MG germs in **A** and **C**. Error bars indicate mean ± SD from at least 20 MGs of total 3 independent experiments. *P < 0.05, **P < 0.01, ***P < 0.001; Student’s *t*-test. Scale bar, 20 μm

To study further the function of PKA and SNAP23 in MG development, we incubated cultured eyelids from EDA^−/−^ mice and evaluated the effects of PKA agonist Forskolin and SNAP23 overexpression (OE). Based on our previous study [14], the average MG length of WT eyelids was 5.6 µm after 2 days of culture. Here, IHC data showed that an EDA supplement largely restored the MG length of EDA^−/−^ eyelids to 4.8 µm, while 20 µM Forskolin only weakly induced MG germs when no EDA was added (Fig. 7A, B). Strikingly, the incubation of EDA together with Forskolin induced ~ 1.46-fold longer MG germs compared to the incubation of EDA alone. The average length of MGs reached 6.9 µm, exceeding the average length of 5.6 µm in WT (Fig. 7A, B). Using AAV5-mediated SNAP23 OE, we observed that SNAP23 OE, like Forskolin, did not significantly restore MG growth in EDA^−/−^ eyelid cultures but sharply augmented the average MG length to 8.3 µm in cultures with EDA treatment (Fig. 7C, D).

Overall, our data suggest that PKA activation and SNAP23 OE, required for the PM trafficking of EDAR, can promote skin appendage growth in an EDA-dependent manner.

## Discussion

It is generally accepted that EDA signaling is the motive force for the etiology and pathogenesis of ectodermal dysplasia (ED) syndromes, given that phenotypically identical conditions can be caused by mutations in *Eda, Edar*, or *Edaradd* genes in a TNF ligand-receptor-adaptor family [8–10]. Similarly, spontaneous mutant mouse strains Tabby, Downless and Crinkled, discovered early in the last century, have been shown to be mutated in the corresponding *Eda, Edar*, and *Edaradd* genes and to have identical “all or none” skin appendage features [8, 10, 27].

**Fig. 8:**
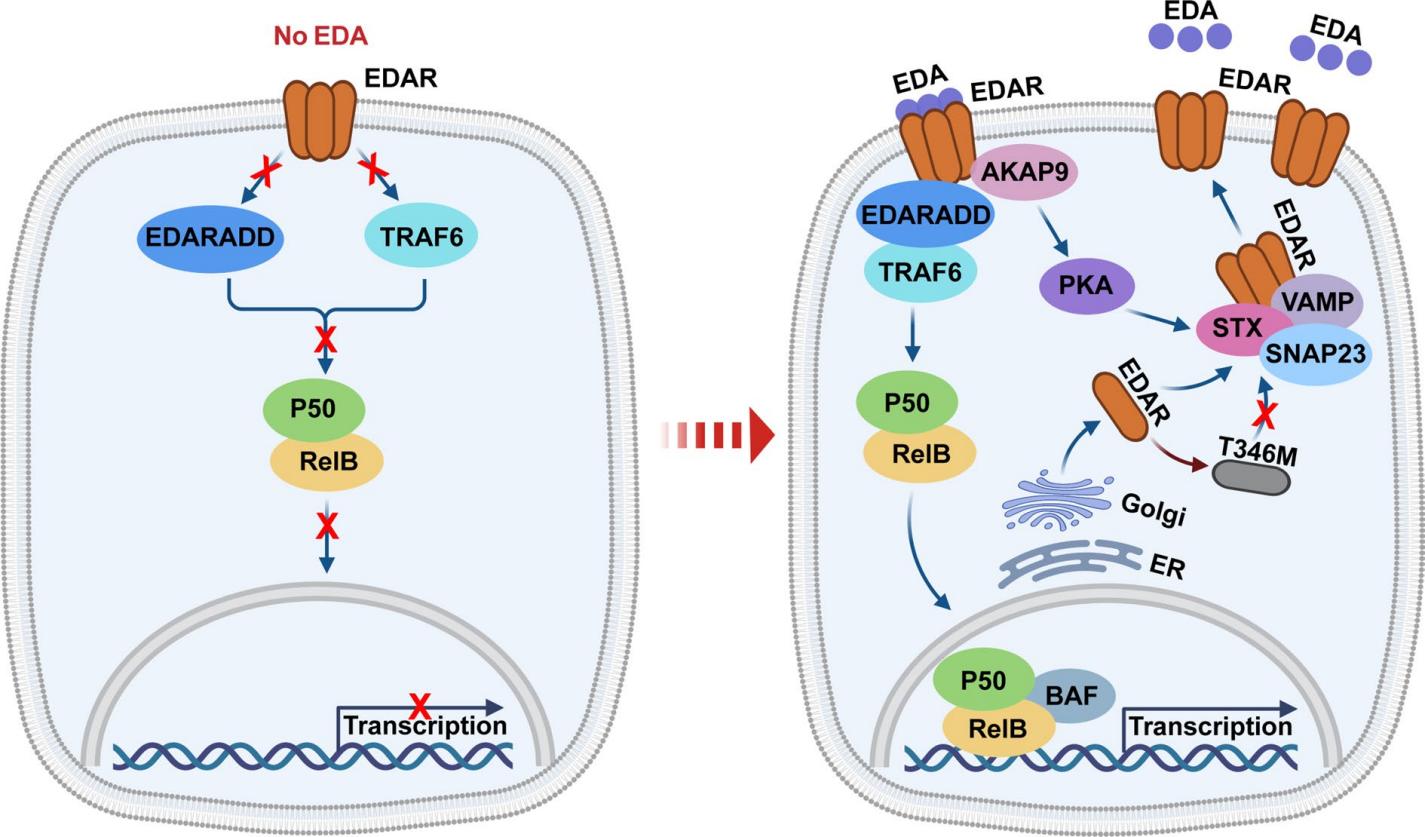
A schematic summary of the present study. The schematic overview shows the mechanism of EDA-induced EDAR plasma membrane trafficking in both cell cultures and skin appendage formation. Our findings indicate that only a small portion of EDAR is located on cell membrane when no EDA appears and EDA signaling is blocked. Here, we find that EDA binding to plasma membrane EDAR initiates a pathway to exacerbate further plasma membrane trafficking of cytosolic EDAR. We also identify a HED-linked EDAR mutation T346M, which leads to the failure of EDAR translocation to plasma membrane. Our study thus provides a model of ligand-triggered plasma membrane trafficking of its own receptor for positive feedback regulation

Like other TNFR members, cell membrane EDAR mainly activates NF-κB for downstream gene expression [4]. Over the past two decades, the mechanism of EDAR-triggered NF-κB activation and many downstream transcriptional targets of NF-κB have been revealed [4, 20]. However, many aspects of EDAR dynamics—including its biosynthesis, post-translational modification and plasma membrane (PM) trafficking—remain to be elucidated. We have studied the EDA-EDAR interaction and find unexpectedly that EDA induces rapid PM translocation of the bulk of its receptor EDAR. Focusing on how EDAR is transported into PM upon EDA stimulation, we delineate three stages in the process: (1) The majority of EDAR is normally maintained in an intracellular cytosolic compartment (Cyt); (2) EDA binds to a small amount of PM EDAR and induces PKA activation; and (3) SNAP23, STX6 and VAMP1/2/3 are recruited to Cyt EDAR and facilitate its trafficking to the PM (Fig. 8).

One might wonder why EDAR pre-exists in an intracellular location when unbound to its ligand EDA. Recent findings have shown that only EDA-unbound cell membrane EDAR in melanoma cells exerts a proapoptotic action by recruiting Caspase-8 [28]. Thus, a possible rationale is that keratinocytes may not allow “more” EDAR on the cell membrane to avoid unwanted cell apoptosis during skin appendage development when there is no EDA expression. Yet, when EDA appears, more PM EDAR is needed for adequate signaling transduction. This possible reciprocal interaction model of EDA-EDAR warrants further investigation.

It is noteworthy that both XEDAR and TNFR1, other TNFR family members, show no such ligand-dependent PM transportation (Fig. 1C–E). To our knowledge, there are only a few reports about their trafficking. Upon binding to ligands, TNFR1 undergoes a clathrin-dependent internalization in some types of cells, functioning as a feedback regulatory mechanism of TNFR signaling [29, 30]. In addition, when binding to TNF ligands, PM TNFR1 can be transported into lipid rafts and has thus been regarded as a prerequisite for association with the TRADD/TRAF2/RIP1 complex and subsequent NF-κB activation [31]. However, studies about the PM transportation of TNFR proteins have not yet been reported for the other more than 25 identified superfamily TNFR proteins. Whether any of them act like EDAR remains an open question.

Most HED cases are X-linked and caused by mutations in EDA genes. But approximately 70 EDAR mutations have also been identified in autosomal dominant or recessive HED patients (Human Gene Mutation Database, https://www.hgmd.cf.ac.uk). Among these mutations, underlying pathogenetic mechanisms for autosomal recessive action have been well documented. Some are predicted to be rapidly degraded and others are shown or predicted to abrogate binding to EDA or EDARADD [25, 32–35]. Interestingly, nearly 10 distinct EDAR mutations have been reported to link to autosomal dominant HED that are either missense or premature stop codon mutations within the death domain of the protein [25, 36–40]. To identify possible mutations that cause defective PM trafficking of EDAR, we selected 6 mutations with strong phenotypes in HED patients for this study; they are all evenly distributed within the intracellular domain to minimize any effect on binding to EDA. Indeed, we find that two mutants T346M and R420W can block the PM translocation of EDAR (Fig. 5). The HED patients with the T346M mutation show a severe condition, with abnormal hair, absence of breasts, dry skin, malformed teeth, and many other features [23]. In line with these clinical findings, our results in MG tissues also indicate that ectopic expression of T346M completely diminishes the growth of MG germs (Fig. 6). They are also likely to have dry eye symptom caused by defective MGs [21]. An R420W mutation we tested in this study has been previously reported to inhibit the interaction between EDAR and EDARADD [22], and our data provide a further alternative pathogenic mechanism of R420W—namely, its ability to impede EDA-dependent PM translocation of EDAR (Fig. 5).

As an obligate part of the mechanism, our results in MG growth from EDA^−/−^ mice demonstrate that either PKA activation or SNAP23 OE can largely promote MG formation when supplementing EDA, and can also mildly restore MG germs even without EDA (Fig. 7). These findings may thus make PKA and SNAP23 targets for novel potential therapeutic intervention for HED.

Overall, our findings reveal a regulatory mechanism of EDA-induced PM trafficking of its receptor EDAR and demonstrate defective EDAR transportation in HED-linked T346M and R420W mutations. However, our study also raises several questions for further investigation: (1) how does PKA activation lead to the recruitment of SNAP23/STX6/VAMP to EDAR? (2) why should the bulk of EDAR be sequestered in the Cyt structure when no EDA is present? and (3) do any other TNFR members share the same regulatory mechanism?

## Conclusions

In sum, we find a novel mechanism of EDA-induced plasma membrane translocation of EDAR, which may exemplify a general model of ligand-triggered cell surface trafficking of its own receptor for positive feedback regulation, and also provide potential targets for HED therapy.

## Supplementary Information

**Additional file 1: **Fig S1. EDA triggers PM translocation of its receptor EDAR. Fig S2. PM protein levels of EDAR increase while Cyt EDAR decrease during 10 to 30 min with EDA treatment. **Fig S3.** EDA does not increase total EDAR protein levels within 2 h. **Fig S4.** EDA triggers PM translocation of its receptor EDAR. **Fig S5.** EDAR-associated proteins are colocalized with EDAR under EDA treatment. **Fig S6.** EDAR-associated protein levels increase in total cell lysate upon EDA treatment. **Fig S7.** SNAP23 siRNA efficiently downregulates SNAP23. **Fig S8.** A schematic shows the structures of AAV plasmids constructed in this study. **Fig S9.** H89 inhibits PKA activity and plasma membrane trafficking of EDAR in skin tissues. **Fig S10.** SNAP23 knockdown inhibits plasma membrane trafficking of EDAR in skin tissues. **Fig S11.** Screening identifies HED-linked mutations in EDAR leading to failed PM translocation. **Table S1.** Components of EDAR complexes determined by MS. **Table S2.** Antibodies used in this study. **Table S3.** The overlapping PCR primers used in site-directed mutagenesis of EDAR.

## Data Availability

Any additional data and materials are available from corresponding authors on reasonable request.

## Abbreviations

AAV: Adeno-associated virus
A/HED: Anhidrotic/hypohidrotic ectodermal dysplasia
EDA: Ectodysplasin-A
Cyt: Cytosolic compartment
PM: Plasma membrane
IF: Immunofluorescence
IHC: Immunohistochemistry
IP: Immunoprecipitation
WT: Wild type
