# Supplementary material for: EDA ligand triggers plasma membrane trafficking of its receptor EDAR via PKA activation and SNAP23-containing complexes
Source: Cell Biosci. 2023 Jul 10;13:128. doi: 10.1186/s13578-023-01082-8 (PMC10334605; doi:10.1186/s13578-023-01082-8)
Supplement: Supplementary file 1 — Additional file 1: Fig S1. EDA triggers PM translocation of its receptor EDAR. Fig S2. PM protein levels of EDAR increase while Cyt EDAR decrease during 10 to 30 min with EDA treatment. Fig S3. EDA does not increase total EDAR protein levels within 2 h. Fig S4. EDA triggers PM translocation of its receptor EDAR. Fig S5. EDAR-associated proteins are colocalized with EDAR under EDA treatment. Fig S6. EDAR-associated protein levels increase in total cell lysate upon EDA treatment. Fig S7. SNAP23 siRNA efficiently downregulates SNAP23. Fig S8. A schematic shows the structures of AAV plasmids constructed in this study. Fig S9. H89 inhibits PKA activity and plasma membrane trafficking of EDAR in skin tissues. Fig S10. SNAP23 knockdown inhibits plasma membrane trafficking of EDAR in skin tissues. Fig S11. Screening identifies HED-linked mutations in EDAR leading to failed PM translocation. Table S1. Components of EDAR complexes determined by MS. Table S2. Antibodies used in this study. Table S3. The overlapping PCR primers used in site-directed mutagenesis of EDAR. [file 13578_2023_1082_MOESM1_ESM.docx]

**Supplementary data**

**EDA ligand triggers plasma membrane trafficking of its receptor EDAR via PKA activation and SNAP23-containing complexes**

Yuyuan Yao^1^, Ruihan Yang^1^, Jian Zhu^2^, David Schlessinger^3^ and Jian Sima^1*^

^1^ School of Basic Medicine and Clinical Pharmacy, China Pharmaceutical University, Nanjing, 210009, China.

^2^ Department of Psychology, Eastern Illinois University, Charleston, IL 61920, USA.

^3^ Laboratory of Genetics and Genomics, NIA/NIH-IRP, 251 Bayview Blvd, Room 10B014, Baltimore, Maryland 21224, USA.

* Correspondence should be addressed to J.S. (E-mail: simajian@cpu.edu.cn)


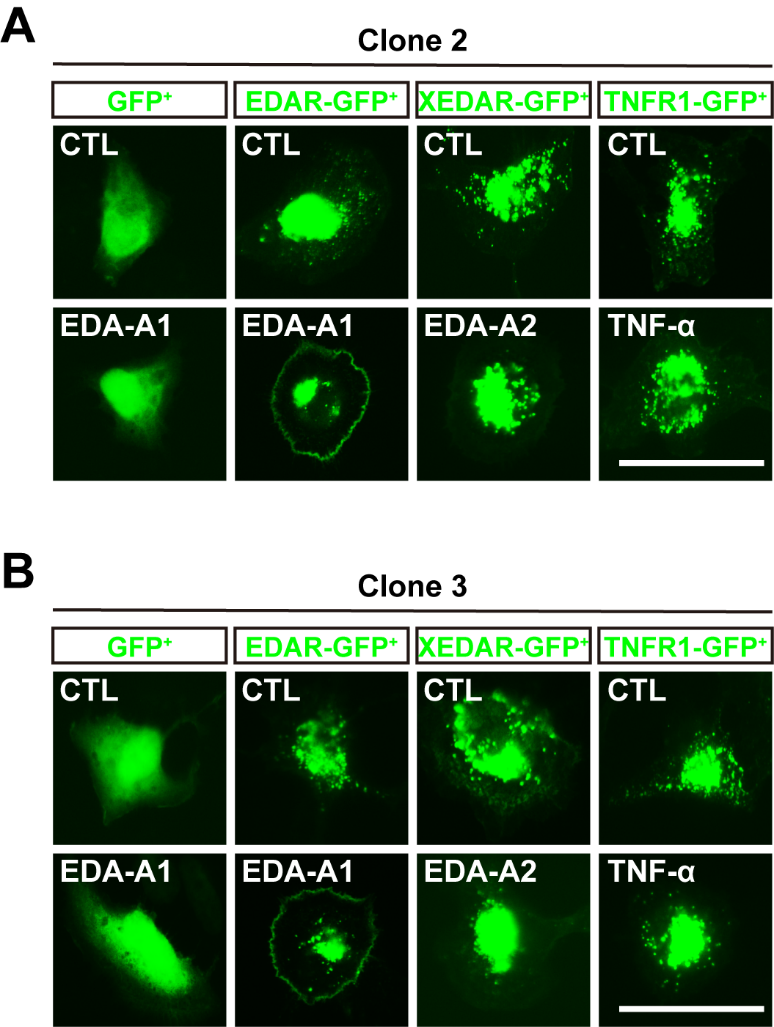


**Supplementary Fig. 1 EDA triggers PM translocation of its receptor EDAR**

As in Fig. 1D, except with other 2 clones of stable cell lines. Scale bar, 20 μm.


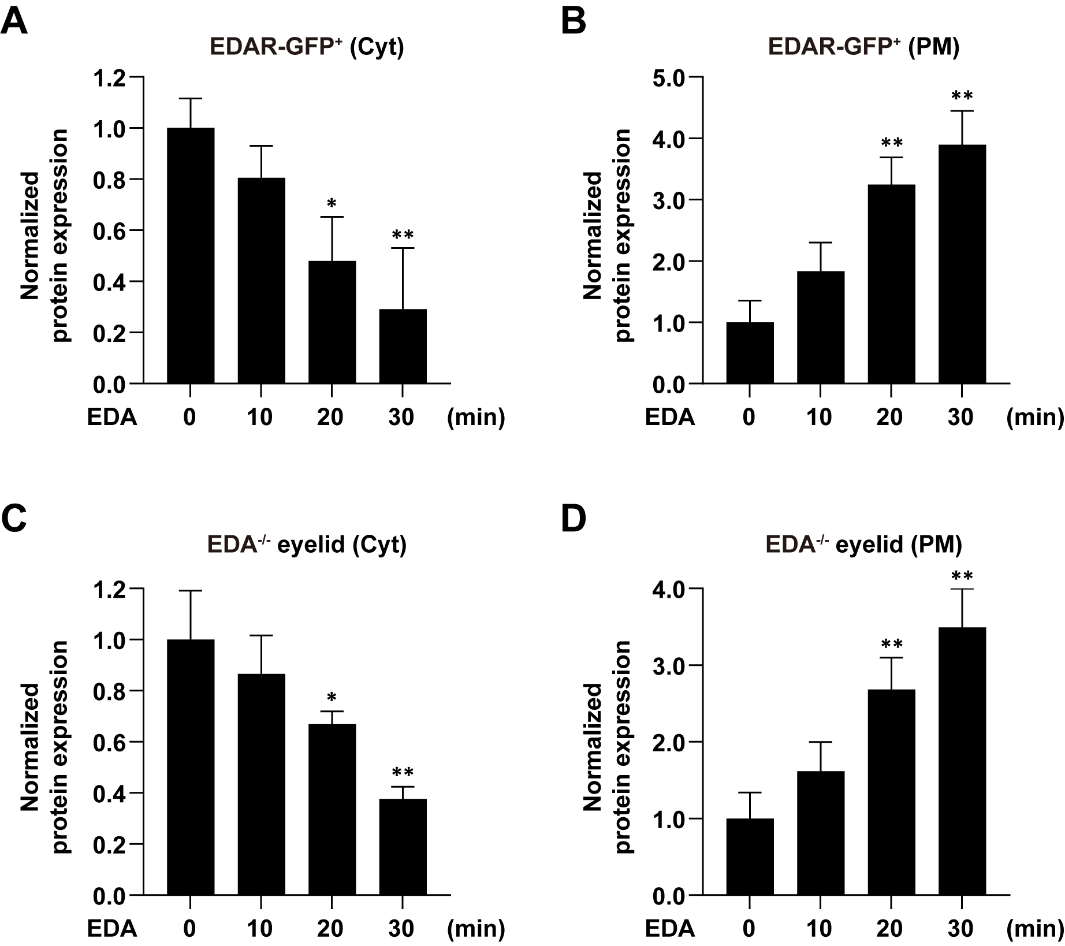


**Supplementary Fig. 2 PM protein levels of EDAR increase while Cyt EDAR decrease during 10 to 30 min with EDA treatment**

Quantification of band intensity in Fig. 1G and Fig. 1H. The protein level at 0 min was normalized to 1.0. Data from 3 independent experiments. Error bars indicate mean ± SD. **P* < 0.05, ***P* < 0.01; Student’s *t*-test.


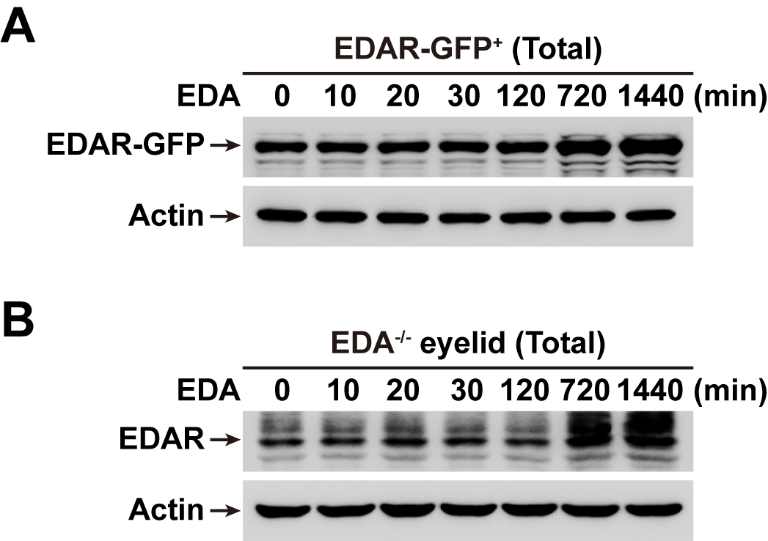


**Supplementary Fig. 3 EDA does not increase total EDAR protein levels within 2 hours**

(A) Immunoblotting shows the levels of total EDAR from EDAR-GFP^+^ cells treated with EDA. Actin serves as a loading control. (B) Similar to (A), except that protein extracts from EDA^-/-^ eyelid cultures were used.


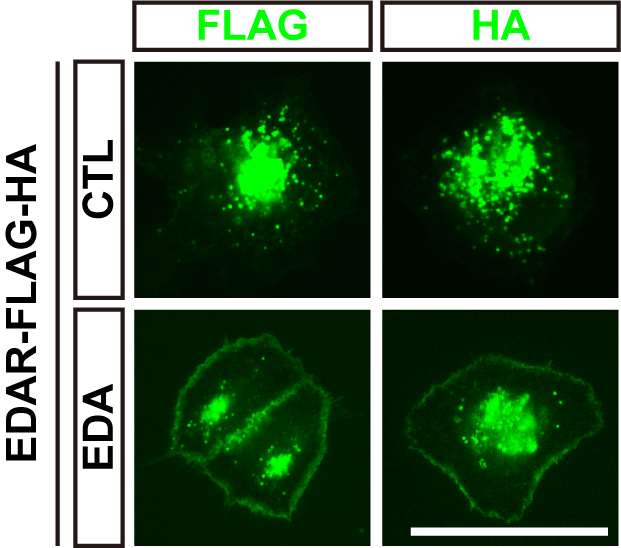


**Supplementary Fig. 4 EDA triggers PM translocation of its receptor EDAR**

Immunofluorescence (IF) images of indicated proteins (green) show the cellular location of EDAR with EDA treatment in EDAR^+^ cells. Scale bar, 20 μm.


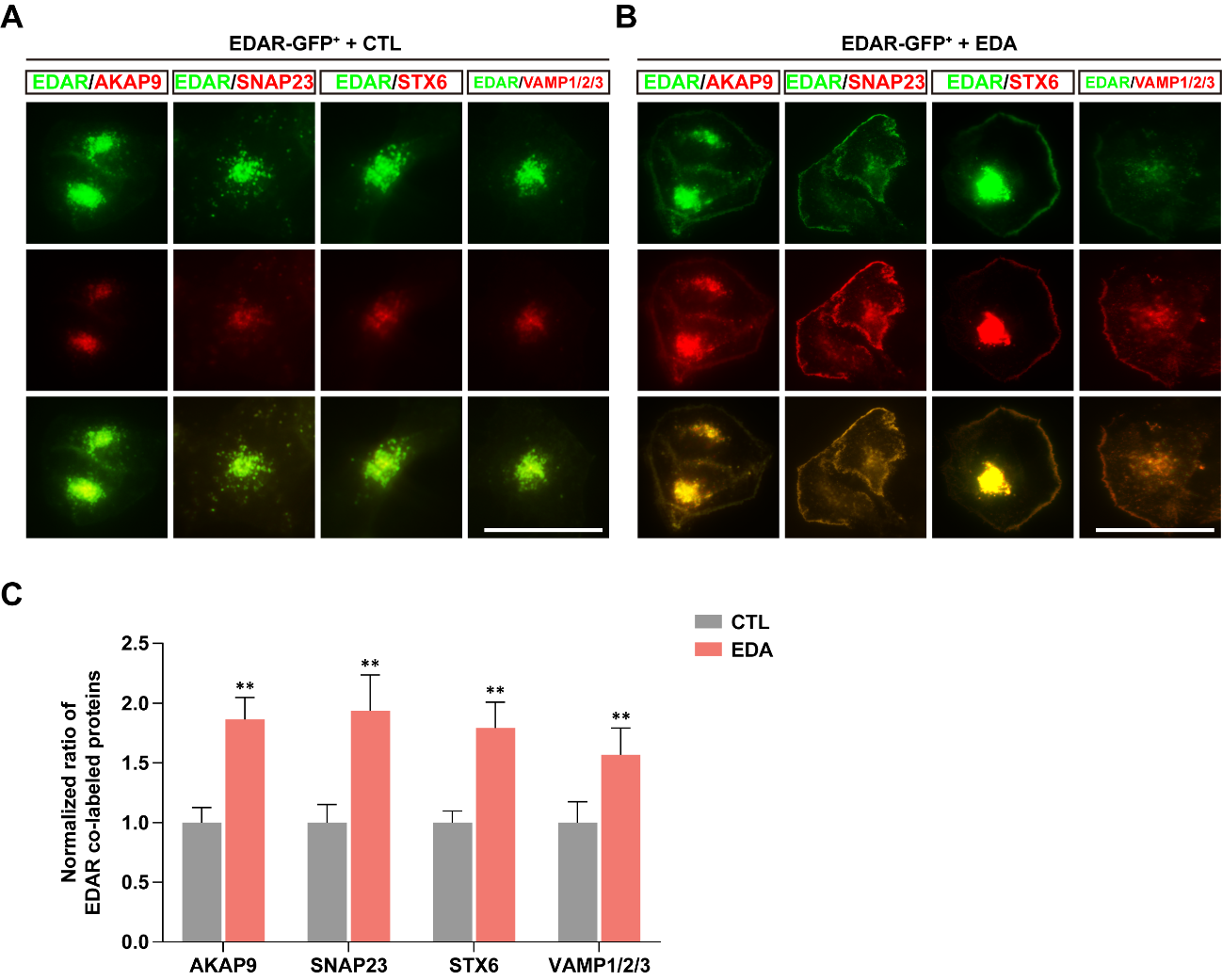


**Supplementary Fig. 5 EDAR-associated proteins are colocalized with EDAR under EDA treatment**

IF images show the subcellular localization of EDAR-associated proteins in EDAR-GFP^+^ cells without (A) or with (B) EDA treatment. Scale bar, 20 μm. (C) Quantitation shows the ratio of EDAR co-labeled with each indicated associated protein in (A) and (B). Error bars indicate mean ± SD from at least 30 cells from 3 independent experiments. ***P* < 0.01; Student’s *t*-test.


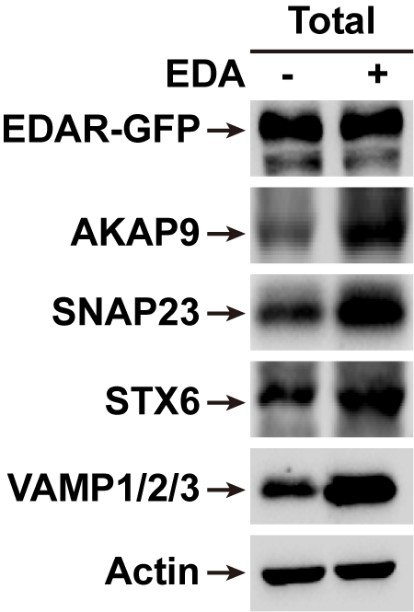


**Supplementary Fig. 6 EDAR-associated protein levels increase in total cell lysate upon EDA treatment**

Immunoblotting shows the levels of indicated proteins in total cell lysate with (+) or without (-) EDA treatment.


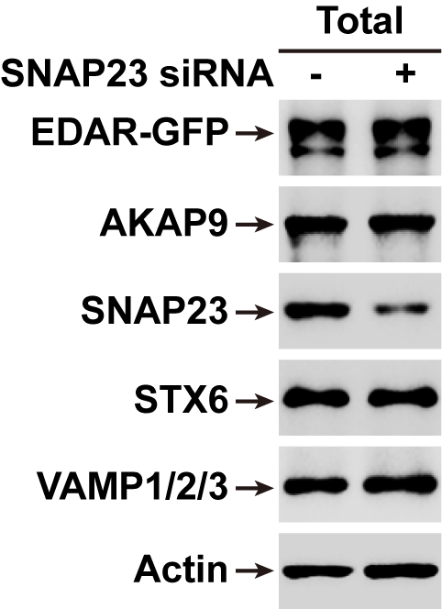


**Supplementary Fig. 7 SNAP23 siRNA efficiently downregulates SNAP23**

Immunoblotting shows the knockdown efficacy of SNAP23 siRNA in EDAR-GFP^+^ cells with no effect on other indicated trafficking proteins.


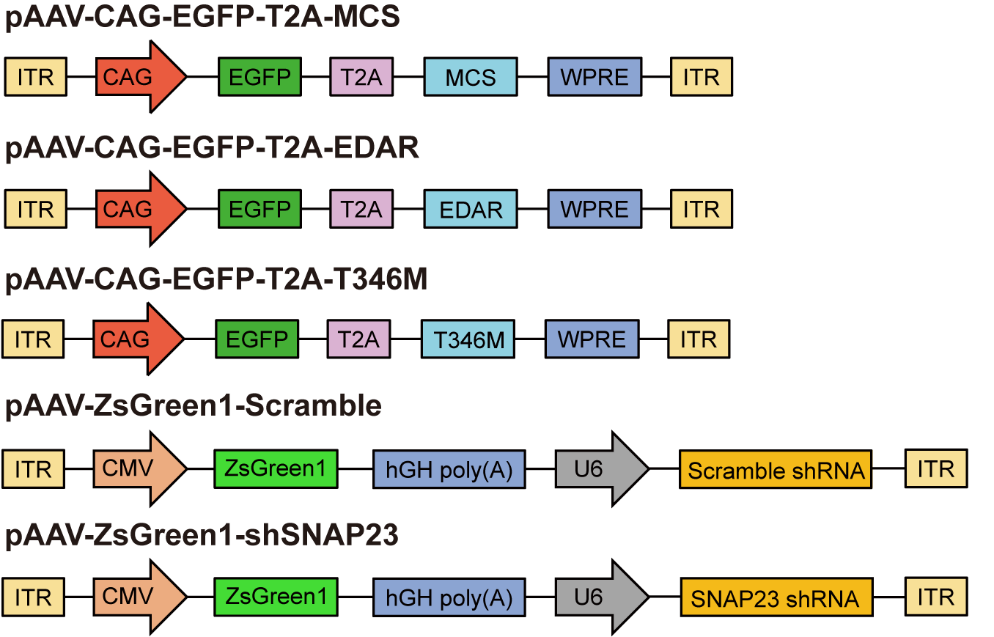


**Supplementary Fig. 8 A schematic shows the structures of AAV plasmids constructed in this study**

Schematic structures of the adeno-associated viral (AAV) plasmids used in this study.


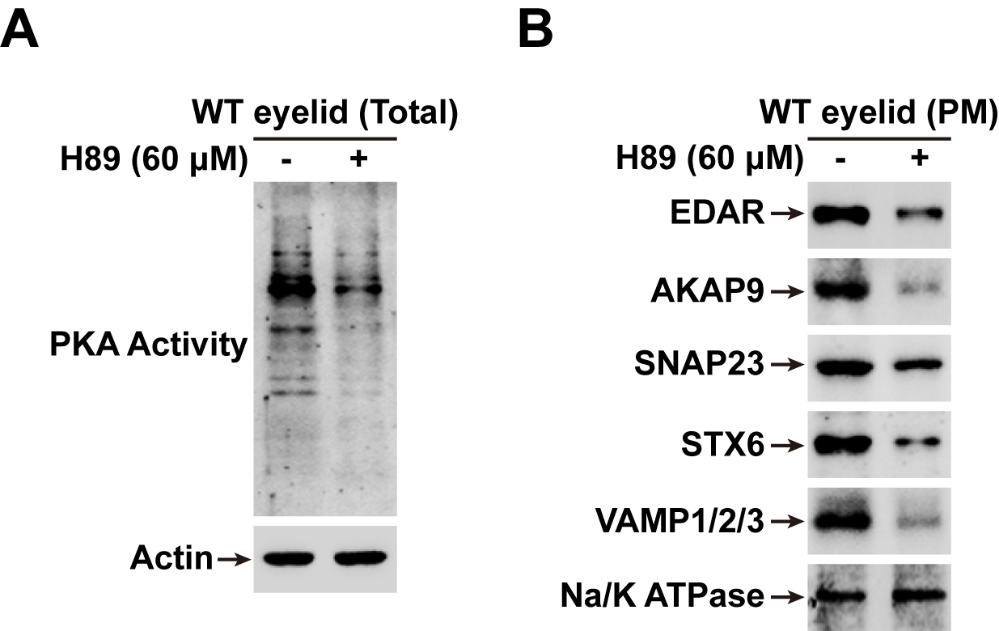


**Supplementary Fig. 9 H89 inhibits PKA activity and plasma membrane trafficking of EDAR in skin tissues**

(A) Immunoblotting shows the intensity of PKA activity in WT eyelid cultures treated with H89 at 60 µM. (B) Immunoblotting shows the levels of indicated proteins in PM fraction from WT eyelid cultures treated with H89.


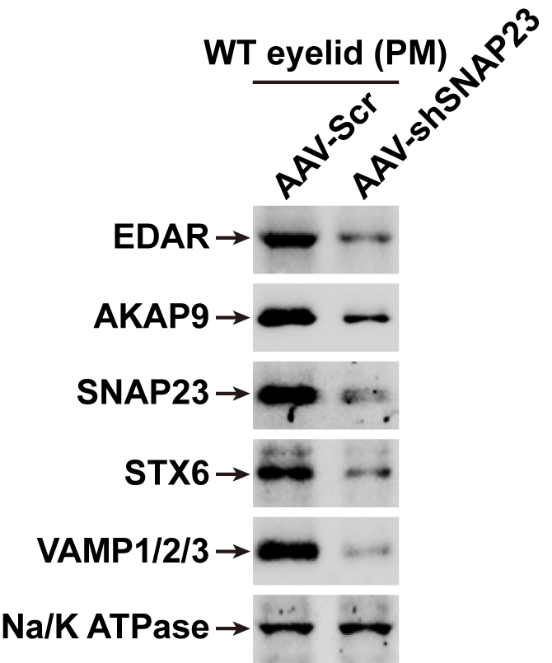


**Supplementary Fig. 10 SNAP23 knockdown inhibits plasma membrane trafficking of EDAR in skin tissues**

Immunoblotting shows the levels of indicated proteins in PM fraction from WT eyelid cultures treated with AAV particles expressing scramble shRNA (left lane) and shSNAP23 (right lane).


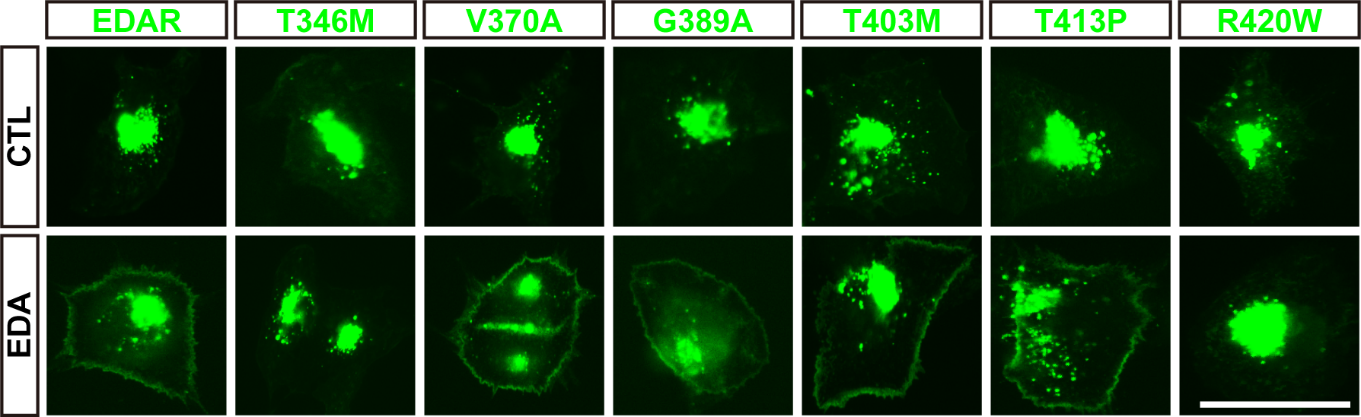


**Supplementary Fig. 11 Screening identifies HED-linked mutations in EDAR leading to failed PM translocation**

Fluorescence images show the subcellular localization of EDAR-GFP in HaCaT cells transfected with plasmids expressing EDAR and each EDAR mutants. Scale bar, 20 μm.

**Supplementary Table 1 Components of EDAR complexes determined by MS**

|  | **FLAG-tag IP** | | **HA-tag IP** | |
| --- | --- | --- | --- | --- |
| **Protein** | **#pept total** | **#pept unique** | **#pept total** | **#pept unique** |
| EDAR | 742 | 28 | 654 | 23 |
| SNAP23 | 46 | 36 | 36 | 24 |
| STX6 | 33 | 24 | 35 | 28 |
| AKAP9 | 29 | 28 | 25 | 25 |
| VAMP1 | 15 | 11 | 20 | 13 |
| VAMP2 | 15 | 10 | 18 | 11 |
| VAMP3 | 15 | 11 | 18 | 12 |
| ZNF585A | 11 | 11 | 12 | 9 |
| PFKP | 11 | 11 | 8 | 8 |
| PTPRD | 11 | 9 | 4 | 4 |
| SMC3 | 11 | 11 | 2 | 2 |
| ATP2B1 | 10 | 9 | 8 | 8 |
| ABCB10 | 10 | 10 | 7 | 7 |
| LSR | 10 | 8 | 7 | 7 |
| STXBP3 | 10 | 10 | 3 | 3 |
| PTPRS | 8 | 8 | 10 | 9 |
| PRKAR2A | 8 | 8 | 3 | 3 |
| SLC3A2 | 7 | 7 | 9 | 8 |
| SLC12A7 | 7 | 7 | 1 | 1 |
| GFPT1 | 6 | 6 | 9 | 9 |
| ACSL3 | 6 | 6 | 6 | 6 |
| SLC20A1 | 6 | 6 | 4 | 4 |
| ALDH1B1 | 6 | 6 | 4 | 4 |
| LRP12 | 6 | 5 | 2 | 2 |
| SLC27A2 | 5 | 5 | 9 | 8 |
| CNNM3 | 5 | 5 | 7 | 6 |
| ADCY3 | 5 | 4 | 7 | 7 |
| RRBP1 | 5 | 5 | 6 | 6 |
| AURKB | 5 | 5 | 6 | 6 |
| VAMP7 | 5 | 5 | 5 | 5 |
| ERMP1 | 5 | 5 | 4 | 3 |
| SLC25A1 | 5 | 5 | 3 | 3 |
| KTN1 | 5 | 5 | 3 | 3 |
| ARL8A | 5 | 5 | 3 | 3 |
| PNPLA6 | 5 | 4 | 3 | 3 |
| ZNF585B | 5 | 5 | 2 | 2 |
| SRPR | 5 | 4 | 2 | 2 |
| NPC1 | 5 | 4 | 2 | 2 |
| MYO6 | 5 | 4 | 1 | 1 |
| NCSTN | 4 | 4 | 7 | 6 |
| DDR1 | 4 | 4 | 7 | 7 |
| FYN | 4 | 4 | 5 | 4 |
| PI4K2A | 4 | 4 | 4 | 3 |
| TMEM205 | 4 | 3 | 4 | 4 |
| TNFRSF10A | 4 | 4 | 3 | 3 |
| TMX4 | 4 | 4 | 3 | 3 |
| STARD3 | 4 | 4 | 3 | 2 |
| CLCN7 | 4 | 4 | 3 | 3 |
| BMPR1A | 4 | 4 | 2 | 2 |
| ACP2 | 4 | 4 | 2 | 2 |
| TGFBR2 | 4 | 4 | 1 | 1 |
| INSR | 4 | 3 | 1 | 1 |
| IARS | 3 | 2 | 9 | 8 |
| HCCS | 3 | 3 | 7 | 5 |
| TNFRSF10B | 3 | 3 | 6 | 4 |
| LEO1 | 3 | 1 | 6 | 1 |
| SCFD1 | 3 | 3 | 5 | 5 |
| LRP10 | 3 | 3 | 5 | 5 |
| ADCY9 | 3 | 3 | 5 | 4 |
| WWP2 | 3 | 3 | 4 | 4 |
| STX4 | 3 | 3 | 4 | 3 |
| RPL4 | 3 | 3 | 4 | 4 |
| SPINT2 | 3 | 2 | 4 | 3 |
| TIMM21 | 3 | 3 | 3 | 3 |
| STX7 | 3 | 3 | 3 | 2 |
| RPL8 | 3 | 3 | 3 | 3 |
| TAP1 | 3 | 3 | 2 | 2 |
| STX12 | 3 | 3 | 2 | 2 |
| TMED3 | 3 | 3 | 1 | 1 |
| CAV1 | 3 | 3 | 1 | 1 |
| VAMP8 | 2 | 2 | 3 | 3 |
| TMEM192 | 2 | 2 | 3 | 3 |
| STOM | 2 | 2 | 3 | 3 |
| SDHB | 2 | 2 | 3 | 3 |
| RAB1A | 2 | 2 | 3 | 2 |
| EGFR | 2 | 2 | 3 | 3 |
| TRAM1 | 2 | 2 | 2 | 2 |
| TMEM55B | 2 | 2 | 2 | 2 |
| CTNNA1 | 1 | 1 | 6 | 5 |
| PCYOX1 | 1 | 1 | 5 | 5 |
| YBX3 | 1 | 1 | 4 | 4 |
| MDN1 | 1 | 1 | 3 | 3 |
| LCLAT1 | 1 | 1 | 3 | 3 |
| ISY1 | 1 | 1 | 3 | 2 |
| GTPBP6 | 1 | 1 | 2 | 2 |
| GPR107 | 1 | 1 | 2 | 1 |
| GNG12 | 1 | 1 | 2 | 2 |
| GNA13 | 1 | 1 | 2 | 2 |
| FIGNL1 | 1 | 1 | 1 | 1 |
| FAM57A | 1 | 1 | 1 | 1 |
| FAM3C | 1 | 1 | 1 | 1 |
| EPHB3 | 1 | 1 | 1 | 1 |
| DCAKD | 1 | 1 | 1 | 1 |
| CSNK1D | 1 | 1 | 1 | 1 |
| CANX | 1 | 1 | 1 | 1 |
| C1orf115 | 1 | 1 | 1 | 1 |
| BTK | 1 | 1 | 1 | 1 |
| BTBD1 | 1 | 1 | 1 | 1 |
| ARMCX3 | 1 | 1 | 1 | 1 |
| AIMP1 | 1 | 1 | 1 | 1 |
| ADRM1 | 1 | 1 | 1 | 1 |
| ABCB11 | 1 | 1 | 1 | 1 |

**Supplementary Table 2 Antibodies used in this study**

| **Antibody** | **Vendor** | **Cat. #** |
| --- | --- | --- |
| Anti-GFP (Nanobody) Agarose Beads | ABclonal | AE074 |
| Goat anti-Mouse IgG (H+L) Secondary Antibody, HRP | Thermo | 31430 |
| Goat anti-Rabbit IgG (H+L) Secondary Antibody, HRP | Thermo | 31460 |
| Goat anti-Rabbit IgG (H+L) Highly Cross-Adsorbed Secondary Antibody, Alexa Fluor 488 | Thermo | A-11034 |
| Goat anti-Rabbit IgG (H+L) Highly Cross-Adsorbed Secondary Antibody, Alexa Fluor 546 | Thermo | A-11035 |
| Goat anti-Mouse IgG (H+L) Highly Cross-Adsorbed Secondary Antibody, Alexa Fluor 488 | Thermo | A-11029 |
| Goat anti-Mouse IgG (H+L) Highly Cross-Adsorbed Secondary Antibody, Alexa Fluor 546 | Thermo | A-11030 |
| Alexa Fluor™ 594 Phalloidin | Thermo | A12381 |
| anti-Keratin K14 guinea pig polyclonal, serum | Progen | GP-CK14 |
| ACTB Rabbit mAb | ABclonal | AC026 |
| Anti-alpha 1 Sodium Potassium ATPase Antibody [464.6] | Abcam | ab7671 |
| Phospho-(Ser/Thr) PKA Substrate Antibody | CST | 9621S |
| AKAP9 Antibody (7E12) | SantaCruz | sc-517030 |
| SNAP23 Antibody | Proteintech | 10825-1-AP |
| Syntaxin 6 (C34B2) Rabbit mAb | CST | 2869S |
| VAMP-1/2/3 Antibody (F-11) | SantaCruz | sc-133129 |
| EDAR Polyclonal Antibody | Proteintech | 18032-1-AP |
| TNFR1 Polyclonal antibody | Proteintech | 21574-1-AP |

**Supplementary Table 3 The overlapping PCR primers used in site-directed mutagenesis of EDAR**

| **Primer name** | **DNA sequence (5’-3’)** |
| --- | --- |
| EDAR-mut T413P-F | TGAGCTACTCCCAAAACTGGT |
| EDAR-mut T413P-R | ACCAGTTTTGGGAGTAGCTCAGG |
| EDAR-mut T403M-F | CTTTGACCGCATCAGCATGGCAGGCTACAGCATC |
| EDAR-mut T403M-R | GATGCTGTAGCCTGCCATGCTGATGCGGTCAAAG |
| EDAR-mut T346M-F | GGTCTTAGCCCCATGGAGCTGCCATTTG |
| EDAR-mut T346M-R | CAAATGGCAGCTCCATGGGGCTAAGACC |
| EDAR-mut V370A-F | AACTCTGAGAAGGCTGCTGTGAAAACGTGGCGC |
| EDAR-mut V370A-R | GCGCCACGTTTTCACAGCAGCCTTCTCAGAGTT |
| EDAR-mut G389A-F | AGAGGGATGAGATTGCGGGCATGACAGACG |
| EDAR-mut G389A-R | CGTCTGTCATGCCCGCAATCTCATCCCTCT |
| EDAR-mut R420W-F | CTGGTGCAGATTGAGTGGCTGGATGCTGTGG |
| EDAR-mut R420W-R | CCACAGCATCCAGCCACTCAATCTGCACCAG |
